# Role of DNA Methylation and Epigenetic Silencing of *HAND2* in Endometrial Cancer Development

**DOI:** 10.1371/journal.pmed.1001551

**Published:** 2013-11-12

**Authors:** Allison Jones, Andrew E. Teschendorff, Quanxi Li, Jane D. Hayward, Athilakshmi Kannan, Tim Mould, James West, Michal Zikan, David Cibula, Heidi Fiegl, Shih-Han Lee, Elisabeth Wik, Richard Hadwin, Rupali Arora, Charlotte Lemech, Henna Turunen, Päivi Pakarinen, Ian J. Jacobs, Helga B. Salvesen, Milan K. Bagchi, Indrani C. Bagchi, Martin Widschwendter

**Affiliations:** 1 Department of Women's Cancer, UCL Elizabeth Garrett Anderson Institute for Women's Health, University College London, London, United Kingdom; 2 Statistical Cancer Genomics, UCL Cancer Institute, University College London, London, United Kingdom; 3 CAS-MPG Partner Institute for Computational Biology, Chinese Academy of Sciences, Shanghai Institute for Biological Sciences, Shanghai, China; 4 Department of Molecular and Integrative Physiology, University of Illinois at Urbana-Champaign, Urbana, Illinois, United States of America; 5 Department of Comparative Biosciences, University of Illinois at Urbana-Champaign, Urbana, Illinois, United States of America; 6 Centre for Mathematics and Physics in the Life Sciences and Experimental Biology, University College London, London, United Kingdom; 7 Gynaecologic Oncology Center, Department of Obstetrics and Gynaecology, First Faculty of Medicine and General University Hospital, Charles University Prague, Prague, Czech Republic; 8 Department of Gynaecology and Obstetrics, Innsbruck Medical University, Innsbruck, Austria; 9 Department of Obstetrics and Gynaecology, Haukeland University Hospital, Bergen, Norway; 10 Center for Cancer Biomarkers, Department of Clinical Science, University of Bergen, Bergen, Norway; 11 Department of Pathology, UCL Cancer Institute, University College London, London, United Kingdom; 12 Department of Medical Oncology, UCL Cancer Institute, University College London, London, United Kingdom; 13 Department of Obstetrics and Gynaecology, Helsinki University Central Hospital, Helsinki, Finland; 14 Faculty of Medical and Human Sciences, University of Manchester, Manchester, United Kingdom; Vanderbilt University, United States of America

## Abstract

**Background:**

Endometrial cancer incidence is continuing to rise in the wake of the current ageing and obesity epidemics. Much of the risk for endometrial cancer development is influenced by the environment and lifestyle. Accumulating evidence suggests that the epigenome serves as the interface between the genome and the environment and that hypermethylation of stem cell polycomb group target genes is an epigenetic hallmark of cancer. The objective of this study was to determine the functional role of epigenetic factors in endometrial cancer development.

**Methods and Findings:**

Epigenome-wide methylation analysis of >27,000 CpG sites in endometrial cancer tissue samples (*n* = 64) and control samples (*n* = 23) revealed that *HAND2* (a gene encoding a transcription factor expressed in the endometrial stroma) is one of the most commonly hypermethylated and silenced genes in endometrial cancer. A novel integrative epigenome-transcriptome-interactome analysis further revealed that *HAND2* is the hub of the most highly ranked differential methylation hotspot in endometrial cancer. These findings were validated using candidate gene methylation analysis in multiple clinical sample sets of tissue samples from a total of 272 additional women. Increased *HAND2* methylation was a feature of premalignant endometrial lesions and was seen to parallel a decrease in RNA and protein levels. Furthermore, women with high endometrial *HAND2* methylation in their premalignant lesions were less likely to respond to progesterone treatment. *HAND2* methylation analysis of endometrial secretions collected using high vaginal swabs taken from women with postmenopausal bleeding specifically identified those patients with early stage endometrial cancer with both high sensitivity and high specificity (receiver operating characteristics area under the curve = 0.91 for stage 1A and 0.97 for higher than stage 1A). Finally, mice harbouring a *Hand2* knock-out specifically in their endometrium were shown to develop precancerous endometrial lesions with increasing age, and these lesions also demonstrated a lack of PTEN expression.

**Conclusions:**

*HAND2* methylation is a common and crucial molecular alteration in endometrial cancer that could potentially be employed as a biomarker for early detection of endometrial cancer and as a predictor of treatment response. The true clinical utility of *HAND2* DNA methylation, however, requires further validation in prospective studies.

*Please see later in the article for the Editors' Summary*

## Abstract 2

TB filled in by Laureen

*Please see later in the article for the Editors' Summary*

## Editors' Summary

**Background:**

Cancer, which is responsible for 13% of global deaths, can develop anywhere in the body, but all cancers are characterized by uncontrolled cell growth and reduced cellular differentiation (the process by which unspecialized cells such as “stem” cells become specialized during development, tissue repair, and normal cell turnover). Genetic alterations—changes in the sequence of nucleotides (DNA's building blocks) in specific genes—are required for this cellular transformation and subsequent cancer development (carcinogenesis). However, recent evidence suggests that epigenetic modifications—reversible, heritable changes in gene function that occur in the absence of nucleotide sequence changes—may also be involved in carcinogenesis. For example, the addition of methyl groups to a set of genes called stem cell polycomb group target genes (PCGTs; polycomb genes control the expression of their target genes by modifying their DNA or associated proteins) is one of the earliest molecular changes in human cancer development, and increasing evidence suggests that hypermethylation of PCGTs is an epigenetic hallmark of cancer.

**Why Was This Study Done?:**

The methylation of PCGTs, which is triggered by age and by environmental factors that are associated with cancer development, reduces cellular differentiation and leads to the accumulation of undifferentiated cells that are susceptible to cancer development. It is unclear, however, whether epigenetic modifications have a causal role in carcinogenesis. Here, the researchers investigate the involvement of epigenetic factors in the development of endometrial (womb) cancer. The risk of endometrial cancer (which affects nearly 50,000 women annually in the United States) is largely determined by environmental and lifestyle factors. Specifically, the risk of this cancer is increased in women in whom estrogen (a hormone that drives cell proliferation in the endometrium) is functionally dominant over progesterone (a hormone that inhibits endometrial proliferation and causes cell differentiation); obese women and women who have taken estrogen-only hormone replacement therapies fall into this category. Thus, endometrial cancer is an ideal model in which to study whether epigenetic mechanisms underlie carcinogenesis.

**What Did the Researchers Do and Find?:**

The researchers collected data on genome-wide DNA methylation at cytosine- and guanine-rich sites in endometrial cancers and normal endometrium and integrated this information with the human interactome and transcriptome (all the physical interactions between proteins and all the genes expressed, respectively, in a cell) using an algorithm called Functional Epigenetic Modules (FEM). This analysis identified *HAND2* as the hub of the most highly ranked differential methylation hotspot in endometrial cancer. *HAND2* is a progesterone-regulated stem cell PCGT. It encodes a transcription factor that is expressed in the endometrial stroma (the connective tissue that lies below the epithelial cells in which most endometrial cancers develop) and that suppresses the production of the growth factors that mediate the growth-inducing effects of estrogen on the endometrial epithelium. The researchers hypothesized, therefore, that epigenetic deregulation of *HAND2* could be a key step in endometrial cancer development. In support of this hypothesis, the researchers report that *HAND2* methylation was increased in premalignant endometrial lesions (cancer-prone, abnormal-looking tissue) compared to normal endometrium, and was associated with suppression of *HAND2* expression. Moreover, a high level of endometrial *HAND2* methylation in premalignant lesions predicted a poor response to progesterone treatment (which stops the growth of some endometrial cancers), and analysis of *HAND2* methylation in endometrial secretions collected from women with postmenopausal bleeding (a symptom of endometrial cancer) accurately identified individuals with early stage endometrial cancer. Finally, mice in which the *Hand2* gene was specifically deleted in the endometrium developed precancerous endometrial lesions with age.

**What Do These Findings Mean?:**

These and other findings identify *HAND2* methylation as a common, key molecular alteration in endometrial cancer. These findings need to be confirmed in more women, and studies are needed to determine the immediate molecular and cellular consequences of *HAND2* silencing in endometrial stromal cells. Nevertheless, these results suggest that *HAND2* methylation could potentially be used as a biomarker for the early detection of endometrial cancer and for predicting treatment response. More generally, these findings support the idea that methylation of *HAND2* (and, by extension, the methylation of other PCGTs) is not a passive epigenetic feature of cancer but is functionally involved in cancer development, and provide a framework for identifying other genes that are epigenetically regulated and functionally important in carcinogenesis.

**Additional Information:**

Please access these websites via the online version of this summary at http://dx.doi.org/10.1371/journal.pmed.1001551

- The US National Cancer Institute provides information on all aspects of cancer and has detailed information about endometrial cancer for patients and professionals (in English and Spanish)
- The not-for-profit organization American Cancer Society provides information on cancer and how it develops and specific information on endometrial cancer (in several languages)
- The UK National Health Service Choices website includes an introduction to cancer, a page on endometrial cancer, and a personal story about endometrial cancer
- The not-for-profit organization Cancer Research UK provides general information about cancer and specific information about endometrial cancer
- Wikipedia has a page on cancer epigenetics (note: Wikipedia is a free online encyclopedia that anyone can edit; available in several languages)
- The Eve Appeal charity that supported this research provides useful information on gynecological cancers

## Introduction

Accumulating evidence suggests that the epigenome serves as the interface between the genome and the environment [1],[2] and that hypermethylation of stem cell polycomb group target genes (PCGTs—targets for chromatin-modifying complexes that transiently suppress expression and temporarily repress cellular differentiation required for development and stem cell renewal) is an epigenetic hallmark of cancer [3],[4]. PCGT methylation is amongst the earliest molecular changes in human carcinogenesis [5]–[7]. Several lines of evidence suggest that methylation of PCGTs, as triggered by environmental factors and age [5],[8], reduces cellular differentiation, leading to an accumulation of undifferentiated cells susceptible to cancer development [7],[9],[10].

Given that endometrial carcinoma risk is largely determined by non-hereditary factors [11],[12], including age, obesity, and reproductive and environmental factors, it is an ideal disease model to study to further our understanding of the epigenetic mechanisms underlying cancer initiation and progression. While oestrogen drives cell proliferation, progesterone inhibits proliferation of the endometrium and causes cell differentiation. Conditions that are associated with a functional dominance of oestrogen over progesterone (obesity, polycystic ovary syndrome, nulliparity, long-term exposure to unopposed oestrogens) increase the risk for endometrial cancer [13].

Although it is well established that the tumour-protective and anti-proliferative effect of progesterone on the endometrial epithelium [14] is mediated via progesterone receptor (PR) activity in the endometrial stroma (and not directly via epithelial PR) [15], very little is known about early molecular changes that contribute to the development of this disease. Here we applied an epigenome-wide approach in conjunction with a novel statistical algorithm to identify genes that are epigenetically silenced early in endometrial carcinogenesis. We validated our findings in multiple clinical sample sets and provide a hypothesis regarding the genetic consequences of epigenetic silencing using a conditional knock-out mouse model.

## Methods

### Analyses Synopsis

We analysed the DNA methylation (DNAme) of ∼27,000 CpGs (Illumina Infinium HumanMethylation27K BeadChip) in normal and cancerous endometrial tissue (Set 1) and applied a novel integrative epigenome-transcriptome-interactome approach (by also adding data from Set 2) to identify epigenetically deregulated interactome hotspots of functional significance associated with the phenotype of interest, i.e., endometrial cancer. The highest scoring gene was *HAND2*, a gene encoding a transcription factor expressed in the endometrial stroma. This result was further validated using real-time PCR, MethyLight, and immunohistochemistry in additional sample sets (Sets 3 and 5). The clinical potential of *HAND2* methylation detection as a marker of early events in the development of endometrial cancer and as a predictor of progesterone treatment response was studied using MethyLight in Sets 4 and 6, respectively. The functional relevance of *HAND2* silencing was addressed through the application of a conditional knock-out mouse model.

### Study Population

#### Set 1 (frozen tissue)

Prospectively collected fresh-frozen tumour tissue donated by consenting patients to a population-based tissue bank at the Haukeland University Hospital, Norway, were analysed. Age at diagnosis of endometrial cancer, International Federation of Gynecology and Obstetrics stage, histological subtype and grade, treatment, and follow-up were registered. The tumour tissue was consecutively examined by frozen sections to verify high malignant epithelial component, with a minimum cutoff for inclusion of 50% purity. Written consent was provided by all patients. 64 endometrial cancer samples and 23 normal endometrial samples from cancer-free women were assessed using the Illumina Infinium HumanMethylation27K BeadChip array (Table S1). All but one of the women were Caucasian. The study was approved by the Regional Committee for Medical and Health Research Ethics, Western Norway (NSD 15501). A total of 34 molecular markers were analysed as previously described [16]–[18].

#### Set 2 (mRNA dataset)

This gene expression dataset included 79 endometrioid stage I endometrial cancers and 12 samples of atrophic endometrium from postmenopausal women, profiled using the Affymetrix Human Genome 133 Plus 2.0 Array (GSE17025) as described in [19]. All samples were collected under full ethical approval.

#### Set 3 (frozen tissue)

118 endometrial cancer samples and 27 control samples were available from the local biobank at the Department of Gynaecology and Obstetrics, Innsbruck Medical University (patients were treated between January 1989 and April 2000). All women providing tissue samples were Caucasian. Sufficient quality and quantity of DNA was extracted from 101 endometrial cancer samples and 24 normal endometrium samples, which were subsequently assessed using MethyLight (Table S2) and quantitative real-time PCR to ascertain *HAND2* DNAme and mRNA expression status, respectively. Written informed consent is not available from all patients; however, in accordance with the Austrian law, the study was approved by the ethical committee of the Innsbruck Medical University (reference number: UN4044-290/4.9) and conducted in accordance with the Declaration of Helsinki. All samples were anonymised to guarantee the protection of privacy before performing the analysis.

#### Set 4 (vaginal swabs)

Vaginal swabs were collected from women who presented with postmenopausal bleeding to University College London Hospital or the five referring hospitals. Swabs were taken prior to hysteroscopy/endometrial biopsy or hysterectomy. A total of 131 consecutive women were recruited and provided written informed consent. Of 131 swab specimens, 80 yielded DNA of sufficient quantity from the collection medium. Of the 80 samples that passed DNA extraction, 48 samples were finally assessed as full clinical information was available at the time for these specimens. 17 women had no endometrial cancer on histology (mean age 65 years). 18 had a stage 1A endometrioid endometrial cancer (1, 11 and 6 had a grade 1, 2 and 3 cancer respectively; mean age 65 years) whilst 13 had an endometrioid endometrial cancer at more advanced stage (8, 3 and 2 had a stage 1B, 2 and 3 cancer, respectively). Of the 13 higher stage cancers, 1, 10 and 2 were grade 1, 2 and 3; mean age 66 years) (Table S3). The study was approved by the Joint University College London/University College London Hospital Committees on the Ethics of Human Research (No 06/Q0502/89).

#### Set 5 (paraffin tissue)

A total of 37 recently archived formalin-fixed paraffin-embedded blocks were retrieved from the pathology archives at the University College London Hospital consisting of four histological subsets: (1) normal endometrium (*n* = 10) from women who had hysterectomy for benign diseases (six for fibroids and four for prolapse, mean age 56.5 y), (2) unaffected normal endometrium from women with complex atypical hyperplasia (CAH) (*n* = 7, mean age 58.7 y), (3) CAH tissue (*n* = 8, mean age 61.3 y), and (4) endometrioid endometrial cancer (*n* = 12, four samples for each of grade 1, 2, and 3 with mean age 56.5 y, 68.8 y, and 68.5 y, respectively). The tissues were analysed by independent gynaecological pathologists, and 2× 0.6-mm punch cores were taken from the formalin-fixed paraffin-embedded samples using a tissue microarrayer prior to DNA extraction. The study was ethically approved by the UCL Cancer Institute and the UCL/UCLH Biobank for Studying Health and Disease (reference number ECNC01.11). Written informed consent was not obtained for these samples, but all samples were anonymised so that patient information was protected and confidentiality preserved in accordance with the UK Human Tissue Act 2004.

#### Set 6 (endometrial biopsies)

74 women who underwent a hysteroscopy and endometrial biopsy between 2009 and 2011 in Prague and between 1999 and 2011 in Helsinki were retrospectively and consecutively selected for this study. All women were treated with progesterone because of a diagnosis of simple, complex, or complex atypical hyperplasia, prior to a follow-up hysteroscopy and second endometrial biopsy 3 months later. From the 74 patients selected, 42 (mean age 56.2 y) provided sufficient DNA from their initial endometrial biopsy (19, 12, and 11 had a simple, complex, and complex atypical hyperplasia, respectively). Sufficient paraffin tissue remained from 34 of the 42 samples for additional immunohistochemical testing. Oral tibolone, dydrogesterone, norethisterone, lynestrenol, and medroxyprogesterone acetate were taken by one, three, four, six, and 24 women, respectively, and four had a progesterone-containing intrauterine device for at least 3 months. Patients were initially treated for 3 months, and the hysteroscopy/biopsy was repeated. If a patient responded (i.e., normal endometrium on histology, *n* = 29), the treatment was stopped in Prague but continued in Helsinki for 3 additional months. No response after 3 months was observed in 13 patients. In the event of lack of response (hyperplasia on histology, but no progression to atypical hyperplasia or cancer), patients were treated for a further 3 months before a repeat biopsy was performed. If there was still no response, as indicated by biopsy at 6 months, a hysterectomy was recommended. Treatment adherence was monitored according to standard clinical practice, i.e., patient assessment. Written informed consent was provided by all patients. The study was approved by the ethical committee of the General University Hospital and Charles University Prague First Faculty of Medicine (No. 1190/07 S-IV) and the Ethics Committee of the Helsinki and Uusimaa Hospital District (approval number 21/13/03/03/2012). Further details are provided in Table S4.

### DNA Methylation Assays

#### DNA extraction and bisulphite modification

Fresh tissue (Sets 1 and 3), vaginal swabs (Set 4), and formalin-fixed paraffin-embedded tissue (Sets 5 and 6) were extracted using the Qiagen DNeasy Blood & Tissue Kit (69506) and the QIAamp DNA Mini Kit (51304). All DNA samples were then bisulphite-modified using the EZ DNA Methylation Kit D5008 (Zymo Research) according to the manufacturer's instructions.

#### Genome-wide DNA methylation analysis

**Figure 1:**
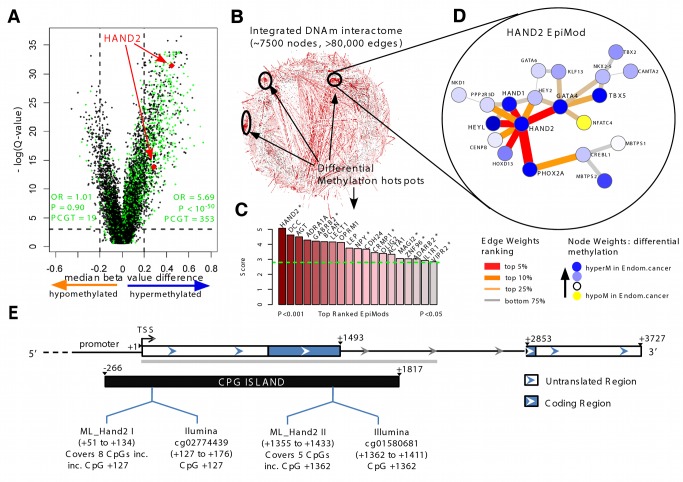
Discovery of *HAND2* methylation as a core feature in endometrial cancer. (A) Volcano plot of epigenome-wide differential DNAme analysis for all 27,578 probes. The *x*-axis indicates the median β-value difference between the normal and cancerous endometrial samples (median[cancer] – median[normal]), while the *y*-axis indicates the −log_e_ scale of *q*-values obtained from a supervised logistic regression analysis testing the association of methylation with normal/cancer status (Set 1). Stem cell PCGT CpGs are highlighted in green, the two *HAND2* CpGs in red. 353 PCGT CpGs are hypermethylated, and 19 PCGT CpGs are hypomethylated, with enrichment odds ratio (OR) and *p*-value (P) obtained from a one-sided Fisher's exact test. The horizontal dotted lines mark the significance cutoffs. (B) Integrative DNA methylome (DNAm)–interactome analysis to identify differential methylation hotspots in the network. Briefly, edge weights in the interactome network reflect the combined differential methylation statistics (absolute values) of the genes making up the edge (the CpG closest to the transcription start site [TSS] of the gene was chosen). A spin-glass module detection algorithm was subsequently used to identify subnetworks where the average edge weight (“modularity”) is higher than random, as assessed by randomly rewiring the network preserving node degrees. Statistical significance of the subnetworks was further assessed by comparing their modularities to those obtained by permuting differential methylation statistics over the network. Subnetworks with *p*<0.05 were called EpiMods. (C) Bar plot of modularity values of the top 19 EpiMods with seed genes as indicated. Asterisks mark those hotspots that remain significant in an integrated DNAme and gene expression interactome analysis, i.e., FEM analysis. (D) The *HAND2* EpiMod. (E) The location of Illumina Infinium HumanMethylation27K array *HAND2* probes and MethyLight reactions (incorporating [inc.] the Illumina HAND2 CpGs) and the sequenced region (grey bar) of *HAND2*. hyperM, hypermethylation; hypoM, hypomethylation.

Genome-wide methylation analysis was performed as described using the validated Illumina Infinium HumanMethylation27K BeadChip [5]. *HAND2* is represented by two CpG sites on the array, as indicated in Figure 1E. The array methylation data are in good agreement with the genes described in the past as hypermethylated in endometrial cancer [20] (Figure S1). The Illumina Infinium HumanMethylation27K DNAme data are available in the Gene Expression Omnibus (accession number GSE40032).

#### *HAND2* MethyLight

MethyLight was performed as previously described [21] using *HAND2*-specific primers and probes: MethyLight *HAND2* I: forward primer: TTAGTTTAGGAGAATTATCGTCGTTATTTC, reverse primer: GAAAACCGCGACTCGAACTC, probe sequence: GAAAACCGCGACTCGAACTC; MethyLight *HAND2* II: forward primer: GATTTTGCGTTTGGTTATTAGTTATATCG, reverse primer: CTCCGCCTCGCCATTCTA, probe sequence: TGGATTTGTTGGTTAAGGACGA. The percentage of methylated reference (PMR) indicates the methylation level at the gene region assessed.

### *HAND2* Sequencing

Sanger sequencing was performed as outlined in Table S5.

### *HAND2* mRNA Expression and Immunohistochemistry

*HAND2* mRNA expression analysis was performed as recently described [22] using the following primers and probe: forward primer: TTTTCTTGTCGTTGCTGCTCA, reverse primer: AAGAAGACCGACGTGAAAGAGG, probe sequence: TTTCAAGATTTCGTTCAGCTCCTTCTTCCTCT. Santa Cruz Biotechnology antibody (SC-9409) was used at a 1∶250 dilution on sections from paraffin-embedded tissue. The Allred Score (total score = proportion score [0–5]+intensity score [0–3]) was used to quantify the results.

### *Hand2* Conditional Knock-Out Mice Experiments

Mice (C57BL/6) were maintained in the designated animal care facility at the University of Illinois College of Veterinary Medicine according to the institutional guidelines for the care and use of laboratory animals. The animals were housed in an Allentown 140 cage IVC system set at 60 air changes per hour at the cage level. The mice were kept on Harlan 1/8 corncob and provided with Harlan 8604 rodent diet. Harlan Iso-blox was provided for environmental enrichment. Rooms were kept on a 12 h/12 h light/dark cycle, and the temperature was set at 72°F with 35%±4% relative humidity. Drinking water was filtered and chlorinated. All animal care, euthanasia, and tissue collection protocols strictly adhered to US National Institutes of Health and institutional guidelines for the use of laboratory animals. All animal protocols were reviewed and approved by the Institutional Animal Use and Care Committee of the University of Illinois. *Hand2* conditional knock-out mice (*Hand2^d/d^*) were generated by crossing PR-Cre knock-in mice with *Hand2* floxed mice (*Hand2^f/f^*) mice as described previously [23]. *Hand2* conditional knock-out females (*Hand2^d/d^*) and the littermate controls (*Hand2^f/f^*) (*n* = 24) were randomly divided into three groups and housed at 2–4 mice per cage. Uterine histology was assessed by hematoxylin and eosin staining or immunohistochemistry at 8–10 (*n* = 5 for each genotype), 24–32 (*n* = 3 for each genotype), and 40–48 wk of age (*n* = 4 for each genotype). Genotyping was performed based on standard protocol using purified mouse tail DNA. *Hand2^f/f^ PR^cre/+^* males and *Hand2^f/f^* females were used as breeding pairs to generate *Hand2* conditional knock-out females (*Hand2^d/d^*) and the corresponding littermate controls (*Hand2^f/f^*). Female mice between 8–10, 24–32, and 40–48 wk of age were euthanized by carbon dioxide inhalation at the designated space in the laboratory. Uterine tissues were excised, trimmed, and collected. Uterine segments were fixed in 10% formalin fixative overnight, then in 75% ethanol for paraffin embedding. Paraffin-embedded endometrial tissue obtained from *Hand2^d/d^* and *Hand2^f/f^* animals were sectioned at 4 µm, mounted on slides, and subjected to immunohistochemistry as described previously [23]. Briefly, uterine sections were incubated at 4°C overnight with polyclonal antibodies against cytokeratin 8 and Ki67. Further incubation was carried out with the biotinylated secondary antibodies at room temperature, followed by incubation with horseradish-peroxidase-conjugated streptavidin (Zymed Laboratories). The sections were stained in AEC Solution (Zymed Laboratories) until optimal signal was obtained. For immunofluorescence the uterine sections were deparaffinised using xylene, and then rehydrated. Antigen retrieval was carried out by boiling the sections in 0.1 M citrate buffer (pH 6.0). Sections were then incubated with normal serum for an hour at room temperature, followed by incubation with the primary antibody overnight at 4°C. Sections were washed in PBS and incubated with secondary antibody linked to fluorochrome for 30 min at room temperature. Sections were washed in PBS and mounted with a coverslip. Negative controls included incubation with normal IgG and omission of the primary antibody (Figure S2). The following primary antibodies were used: PTEN (Millipore, catalog #04-035, 1∶200), p-FRS2 (R&D Systems, catalog #AF 5126, 1∶200), and p53 (Santa Cruz Biotechnology, catalog #SC-6243, 1∶100); secondary antibody for immunofluorescence was from Jackson ImmunoResearch.

### Statistical Analyses

A logistic regression approach was used to model the association between endometrial cancer status (cancer versus normal) and the CpG β-value methylation profile. *p*-Values were estimated using likelihood ratio tests. To correct for multiple testing, we estimated the false discovery rate using the *q*-value estimation procedure [24].

Gene Set Enrichment Analysis (GSEA) is a statistical procedure used to test the hypothesis that a ranked list of genes is enriched for specific biological terms or molecular pathways [25]. As a database of biological terms and pathways (over 6,000 biological terms) we used a recent version of MSigDB Molecular Signatures Database (version 3.0). We also used a term annotated as PolyComb Group Targets from Lee et al [26]. GSEA was performed by computing enrichment odds ratios, with statistical significance estimated using a one-tailed Fisher's exact test. *p*-Values from this test were corrected for multiple testing using the Benjamini-Hochberg procedure [27]. GSEA was performed separately for top-ranked hypermethylated and hypomethylated CpGs, and at the gene level in order to avoid overcounting multiple CpGs mapping to the same gene.

The Functional Epigenetic Modules (FEM) algorithm is a novel direct extension of the EpiMod algorithm developed by us previously [28]. Full details can be found in Text S1. Briefly, it is an integrative epigenome-transcriptome-interactome approach that aims to identify epigenetically deregulated interactome hotspots of functional significance associated with a phenotype of interest, here, endometrial cancer. There are two main steps to the algorithm (see Figure S3). First, DNAme levels of gene promoter regions are integrated with a human interactome to identify differential methylation hotspots associated with endometrial cancer. A differential methylation hotspot represents a closely connected subnetwork of gene promoters whose genes interact at the protein level and for which a significant number of gene promoters are differentially methylated in endometrial cancer. These hotspots (or epigenetic modules [EpiMods]) are identified using a powerful module detection algorithm as described and validated by us in detail in [28]. Second, to assess functional significance, the inference of modules is repeated by further integration with mRNA expression data (full details provided in Text S1). Specifically, the algorithm detects modules that are deregulated at both the DNAme and mRNA expression levels, and in a manner that is consistent with the expected anti-correlation between promoter DNAme and mRNA expression. Modules that are robust and remain significant under the integrated mRNA expression and DNAme analysis are deemed candidate functional EpiMods. The EpiMod and FEM algorithms are freely available as executable R-scripts from http://code.google.com/p/epimods/downloads/list.

The performance of the diagnostic test to distinguish endometrial cancers from non-cancers is assessed by computing the sensitivity and specificity across a range of thresholds, thus generating receiver operating characteristic (ROC) curves, representing plots of sensitivity (*y*-axis) against 1 – specificity (*x*-axis). The area under the ROC curve (AUC) represents a threshold-independent measure of how well the test can discriminate the two phenotypes. The asymptotic confidence interval of the AUC and non-parametric hypothesis-testing *p*-values were calculated using SPSS Statistics version 21 (IBM).

The numerical values prepared from at least three independent samples of mice were analysed by *t*-test when comparisons were made between control and experimental groups (GraphPad Prism 4.0, GraphPad Software). Data are expressed as mean ± standard error of the mean.

## Results

### *HAND2* Is the Top-Ranked Differential Methylation Interactome Hotspot in Endometrial Cancer

We performed DNAme profiling of 27,578 CpGs in 23 normal and 64 cancerous endometrial samples (Set 1; Table S1). Principal component analysis demonstrated that the top component, accounting for over 90% of the variation in the data, was strongly associated with DNAme differences between normal and cancerous endometrium (Wilcoxon rank sum test *p*<10^−10^; Figure S4). Using logistic regressions and adopting a *q*-value (false discovery rate) threshold of <0.1, we identified 2,347 CpGs that were hypermethylated (Table S6), and 1,024 CpGs that were hypomethylated in cases versus controls (Figure 1A). GSEA demonstrated very strong enrichment of EED, SUZ12, H3K27me3, and PRC2 targets, demonstrating that PCGTs are preferentially methylated in endometrial cancer (Figure 1A; odds ratio 5.69 [95% CI 4.91–6.60], *p*<10^−50^; see also Tables S7 and S8).

In order to identify pathways and gene modules that are aberrantly regulated at the epigenetic level, we developed a novel integrative epigenome-transcriptome-interactome approach that infers differential methylation interactome hotspots of functional significance (Figure S3). The resulting algorithm, FEM, is an extension of the EpiMod algorithm developed by us previously [28]. The EpiMod algorithm was extensively tested and validated on independent data and shown to outperform other competing module detection algorithms [28]. Briefly, the algorithm integrates the DNAme data with a human protein interactome to identify gene promoters, not only according to their level of differential methylation in cancer, but also according to whether they define differential methylation interactome hotspots (EpiMods) (Figure 1B). These EpiMods constitute densely connected subnetworks where a significant number of gene promoters exhibit differential methylation. FEM extends the EpiMod algorithm by incorporating gene expression data in an integrative analysis that aims to identify EpiMods that are also functionally deregulated (Figure S3).

We identified a total of 19 significant EpiMods, with *HAND2* emerging as the hub of the top-ranked EpiMod (Figure 1C and 1D; Tables S9 and S10). Importantly, the two CpG probes on the Illumina Infinium HumanMethylation27K BeadChip array mapping to *HAND2* ranked highly among all hypermethylated CpGs (Figure 1A and 1E; Table S6). Furthermore, the *HAND2* EpiMod was a significantly functionally deregulated hotspot under the FEM analysis that incorporated independent gene expression data (12 normal and 79 cancerous endometrial samples—Set 2 [19]) (Figure 1C), with *HAND2* demonstrating concordant underexpression in endometrial cancer (Figure S5). All EpiMods demonstrated strong enrichment for biological terms (Table S11), with the *HAND2* EpiMod itself highly enriched for other transcription factors (e.g., *GATA4, HEY2, HOXD13, PHOX2A, HAND1*), all of which were also hypermethylated in endometrial cancer (Figure S6). All these results indicate that *HAND2* and the interaction neighbourhood of *HAND2*, including *GATA4*, *HEYL*, and *PHOX2A*, represent a core component that is epigenetically deregulated in endometrial cancer. Interestingly, the hub of the second top-ranked EpiMod was *DCC*, a putative tumour suppressor [29]. However, although DNAme of *DCC* strongly correlated with that of *HAND2* (Figure S7), the *DCC* EpiMod did not represent a functionally deregulated hotspot (Figure 1C). Consequently, our novel bioinformatic analysis led us to further investigate the role of *HAND2* in endometrial cancer.

The relevance of *HAND2* in endometrial cancer is supported by several lines of published evidence. First, *HAND2* is a basic helix-loop-helix transcription factor and developmental regulator [30], as well as a stem cell PCGT [26]. Second, it is expressed in the normal endometrial stroma, with its key physiological function being to suppress the production of fibroblast growth factors that mediate the paracrine mitogenic effects of oestrogen on the endometrial epithelium [23]. Finally, *HAND2* is regulated by progesterone [31],[32] and is integral for the progesterone-mediated suppression of oestrogen-induced pathways, with the absence of *HAND2* resulting in impaired implantation [23]. Given this evidence, we postulated that epigenetic deregulation of *HAND2* could represent a key step in endometrial carcinogenesis.

### *HAND2* Methylation Is Associated with HAND2 Suppression

**Figure 2:**
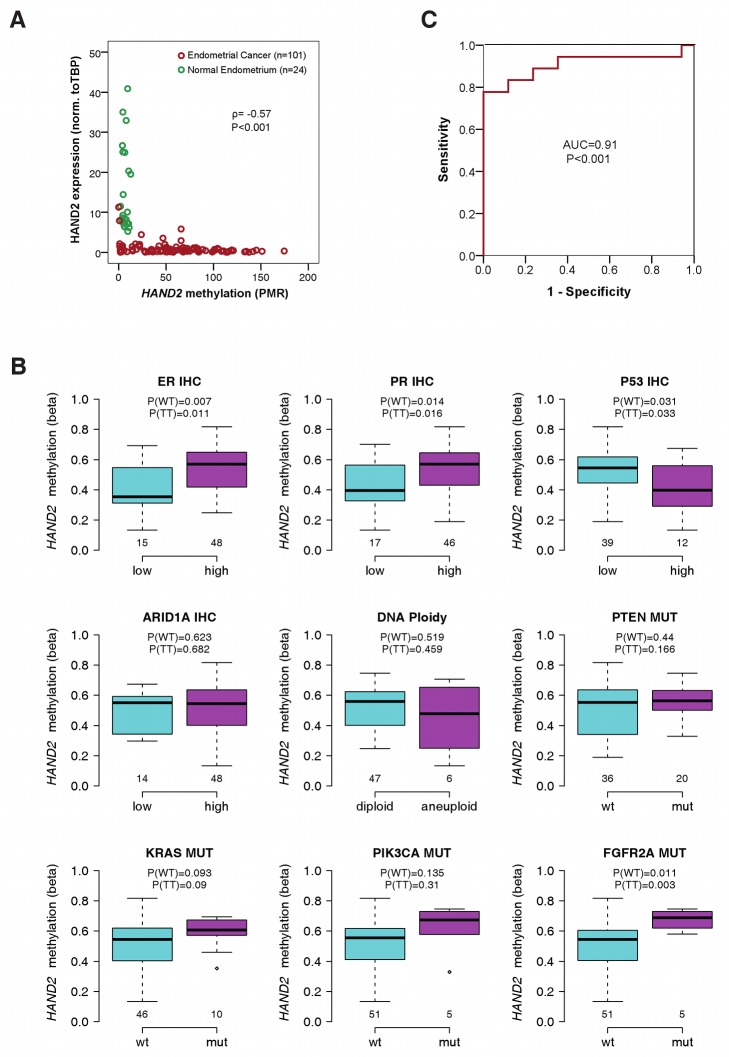
Association of *HAND2* methylation with molecular and clinical features in invasive endometrial cancer. (A) Scatter plot of the MethyLight PMR profile and the gene expression profile of sample Set 3 showing 24 normal endometrium samples (in green) and 101 endometrial cancer samples (in red). The correlation between the two profiles was tested by the Spearman's rank correlation test with the correlation coefficient ρ and corresponding *p*-value (P). (B) Analysis of 34 molecular factors and association with *HAND2* methylation (Set1; Table S12). The nine molecular cancer subgroups with significant heterogeneity between samples are displayed, and the *p*-values for the Wilcoxon rank sum test (WT) and *t*-test (TT) are provided separately. Boxes are median (interquartile range), and whiskers indicate range. (C) ROC curves measuring the sensitivity and specificity of *HAND2* methylation in vaginal swabs to discriminate women with stage 1A endometrial cancer (*n* = 18) from women with non-cancerous causes (*n* = 17) for postmenopausal bleeding (Set 4). PMR values as continuous variables were used for the analysis. AUC and *p*-values (P) as specified. IHC, immunohistochemistry; mut, mutation.

We decided to first validate our array-based data using MethyLight, an alternative real-time-PCR-based assay to study DNAme, in a reaction spanning 5–7 linked CpGs, designed to include those CpGs representing *HAND2* on the Illumina array (Figure 1E). MethyLight was carried out in an additional independent set (Set 3) of endometrial cancers (*n* = 101) and 24 normal endometrial samples from cancer-free women (Table S2). There was a noticeable correlation between the methylation status of the two individual Illumina CpG assays as well as between the two MethyLight reactions incorporating these assays (Figure S8). The MethyLight data confirmed a significantly higher methylation in endometrial cancer samples (Figure 2A).

To validate the impact of *HAND2* methylation on mRNA expression, we measured *HAND2* mRNA expression in the samples of Set 3. Almost all endometrial cancer samples were strongly methylated and demonstrated a significant suppression of *HAND2* mRNA (*p*<0.001; Figure 2A). In contrast, all of the normal endometrial samples demonstrated low DNAme levels and correspondingly high mRNA expression levels (Figure 2A).

### *HAND2* Methylation Is the Most Common Molecular Alteration in Endometrial Cancer

To assess the relative importance of *HAND2* methylation in human endometrial carcinogenesis, we tested for any associations between *HAND2* methylation and well-known molecular characteristics of the tumours (Table S12). Aside from subtle associations of *HAND2* methylation with oestrogen and progesterone receptor immunohistochemistry, p53 expression, and *FGFR2A* mutation status, none of the 30 (Table S12; Figure 2B) remaining molecular features we tested exhibited an association with *HAND2* methylation. Furthermore, the quantitative difference in *HAND2* methylation between normal and cancer tissue was significantly greater than the differences observed between molecular cancer subgroups (compare Figure 2A and 2B). Most importantly, *HAND2* methylation was observed in over 90% of endometrial cancers (Figure S6), and thus represented, by far, the most frequent molecular alteration. Sequencing of the 5′ region of *HAND2* (see Figure 1E) further excluded local changes in DNA sequence as a trigger of *HAND2* DNAme (Table S5; Figure S9).

Finally, *HAND2* DNAme was not associated with any clinicopathological features including grade, stage, and histology, or with clinical outcome in Set 1 (Table S1). In Set 3 there was no association with histology or outcome, but we did observe a trend towards higher methylation in lower stage and lower grade cancers (Table S2). Thus, we can conclude that *HAND2* methylation is a common feature of endometrial cancer, largely independent of sequence variants, clinicopathological characteristics, and specific molecular endometrial cancer subgroups.

### *HAND2* Methylation in Vaginal Fluid Allows for Early Detection of Endometrial Cancer

As DNAme analysis is amenable to assessment in bodily fluids, we investigated the potential diagnostic utility of *HAND2* methylation to identify women with suspected endometrial cancer because of presentation with postmenopausal bleeding. We prospectively collected high vaginal swabs to sample DNA that had drained from the endometrial cavity from (1) 18 women later confirmed to have a stage 1A endometrial cancer, (2) 13 women with an endometrial cancer at more advanced stage, and (3) 17 women who were cancer-free (Set 4). We performed MethyLight of *HAND2* DNAme and calculated the AUC to assess the sensitivity and specificity of the test: the AUC values were 0.91 and 0.97 for stage 1A (Figure 2C) and higher than stage 1A (Figure S10) endometrial cancers, respectively.

### *HAND2* Methylation Is an Early Event in Endometrial Carcinogenesis

**Figure 3:**
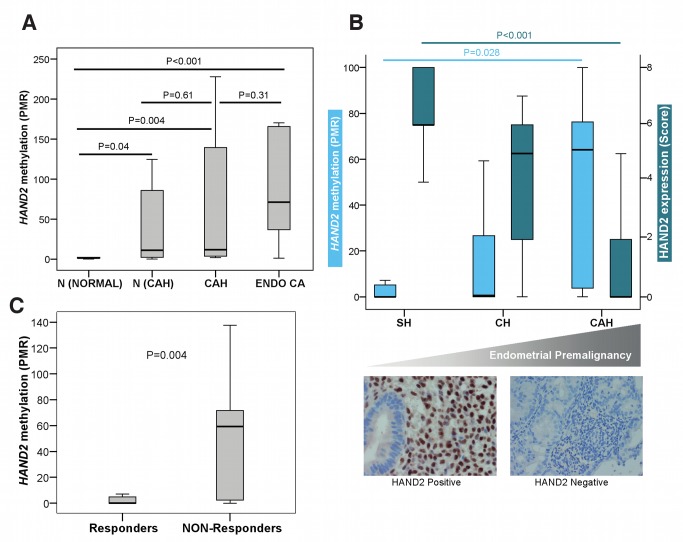
*HAND2* methylation in human endometrial carcinogenesis. (A) Boxplot comparing the differences in the *HAND2* MethyLight PMR profiles in endometrial samples from women without any endometrial pathology (N [Normal], *n* = 10), in normal endometrium from women with CAH (N [CAH], *n* = 7), in CAH samples (CAH, *n* = 8), and in endometrioid endometrial cancer samples (Endo CA, *n* = 12). (B) Boxplots (top panel) comparing DNAme (MethyLight) and HAND2 protein expression (immunohistochemistry quantified by means of the Allred Score) in three different endometrial conditions with increasing potential for malignant transformation (simple hyperplasia [SH], *n* = 17; complex hyperplasia [CH], *n* = 10; CAH, *n* = 7). The lower panel gives an example of the corresponding HAND2 nuclear protein expression in stromal cells in simple hyperplasia compared to loss of stromal expression in CAH. (C) Boxplot comparing the differences in the *HAND2* MethyLight PMR profiles in endometrial cancer from patients treated with progesterone according to whether they had clinically responded (*n* = 29) or were non-responsive (*n* = 13) to treatment. All *p*-values were obtained from the two-sided Wilcoxon rank sum test. Boxes are median (interquartile range), and whiskers indicate range.

As *HAND2* DNAme was a confirmed feature of endometrial cancer, we next sought to determine at which point during endometrial carcinogenesis *HAND2* becomes aberrantly methylated. We measured *HAND2* DNAme in an endometrial cancer progression series (Set 5) including (1) histologically normal endometrium from women without any endometrial pathology, (2) adjacent histologically normal endometrium from women who had areas of CAH elsewhere in their endometrium, (3) CAH lesions, and (4) invasive endometrioid endometrial cancer tissue samples. *HAND2* DNAme in the normal endometrium from women without endometrial pathology was virtually undetectable but was significantly increased in normal endometrium samples from women with CAH, and further still in both CAH lesions and cancerous endometrial tissue (Figure 3A). Importantly, *HAND2* DNAme analysis was able to discriminate normal endometrial tissue from confirmed CAH cases versus normal endometrial tissue from healthy controls: AUC of 0.80 (*p* = 0.04).

### *HAND2* Methylation in Hyperplastic Endometrium Predicts Response to Progesterone

Although we have clearly shown that *HAND2* methylation precedes the development of endometrial cancer, it is still unclear whether epigenetic silencing of *HAND2* can be functionally linked to endometrial cancer development. The sole way to demonstrate this in humans is to test whether activation of *HAND2* expression (via its upstream regulator progesterone) is associated with a change of endometrial histology. If *HAND2* is silenced by DNAme, one would expect no effect of progesterone. As progesterone response is reliant upon *HAND2* expression in the endometrium [23], we examined whether *HAND2* methylation in non-cancerous hyperplasia of the endometrium is functionally associated with response to progesterone treatment, typically administered as an alternative to hysterectomy. We assessed 42 pre-treatment endometrial biopsy samples (Set 6) and observed a significant increase of *HAND2* methylation, and concurrent decrease of HAND2 protein expression, with premalignant disease progression (Figure 3B). As expected, HAND2 protein expression was confined to the stroma and not the glandular epithelium in both normal and simple hyperplastic tissue, which demonstrated very low *HAND2* DNAme despite an increase in the glandular/stromal ratio. Furthermore, we observed that *HAND2* methylation levels were significantly higher in women who did not respond to a 3-mo progesterone treatment period compared to women whose endometrial lesions regressed after treatment (Figure 3C). The AUC of *HAND2* methylation to predict lack of response to progesterone was 0.77 (*p* = 0.005).

### Conditional Knock-Out of *Hand2* in Uterine Tissue Leads to Complex Atypical Hyperplasia as a Function of Age

**Figure 4:**
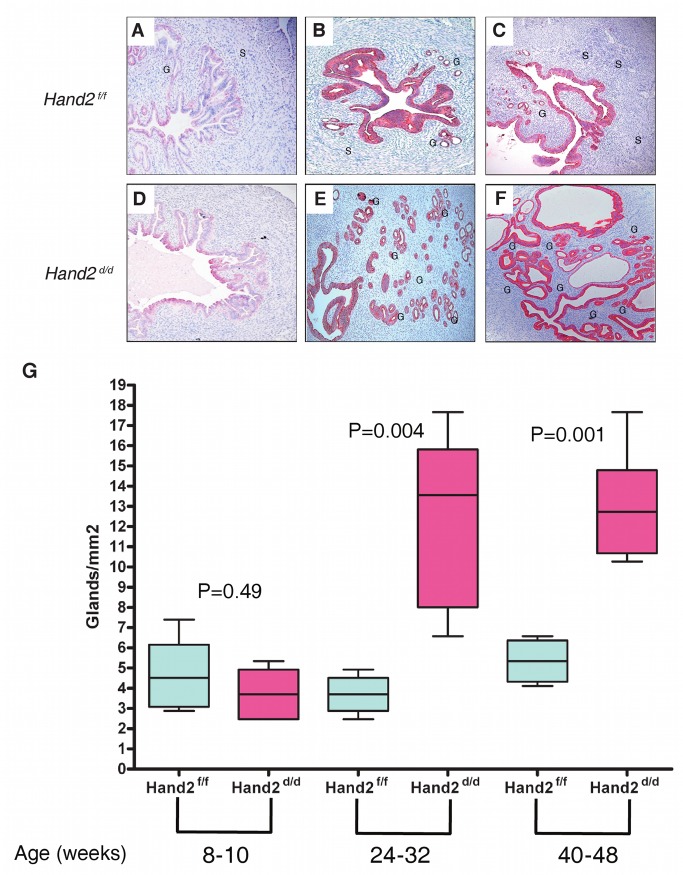
Endometrial hyperplasia in *Hand2* conditional knock-out mice as a function of age. (A–F) Sections of uteri obtained from *Hand2^f/f^* (control) and *Hand2^d/d^* (null) mice were subjected to immunohistochemical staining with an antibody to cytokeratin 8, which marks the epithelial cells. Uterine sections were collected from mice of both genotypes at different ages. Representative uterine sections obtained from mice (*n* = 12) at 8–10 (A and D), 24–32 (B and E), and 40–48 (C and F) wk of age are shown. Note the increase in the gland/stroma ratio and the irregularity in the shape and size of the glands in *Hand2^d/d^* uteri compared to *Hand2^f/f^* uteri. G, gland; S, stroma. Magnification 20×. (G) Boxplots comparing the number of glands in uterine sections of *Hand2^f/f^* (control) and *Hand2^d/d^* (null) mice as a function of age. The number of endometrial glands was determined by counting the glands from three different regions of the uterine horn and is expressed as mean ± standard error. Statistical analysis was performed using a *t*-test. Boxes are median (interquartile range), and whiskers indicate range.

**Figure 5:**
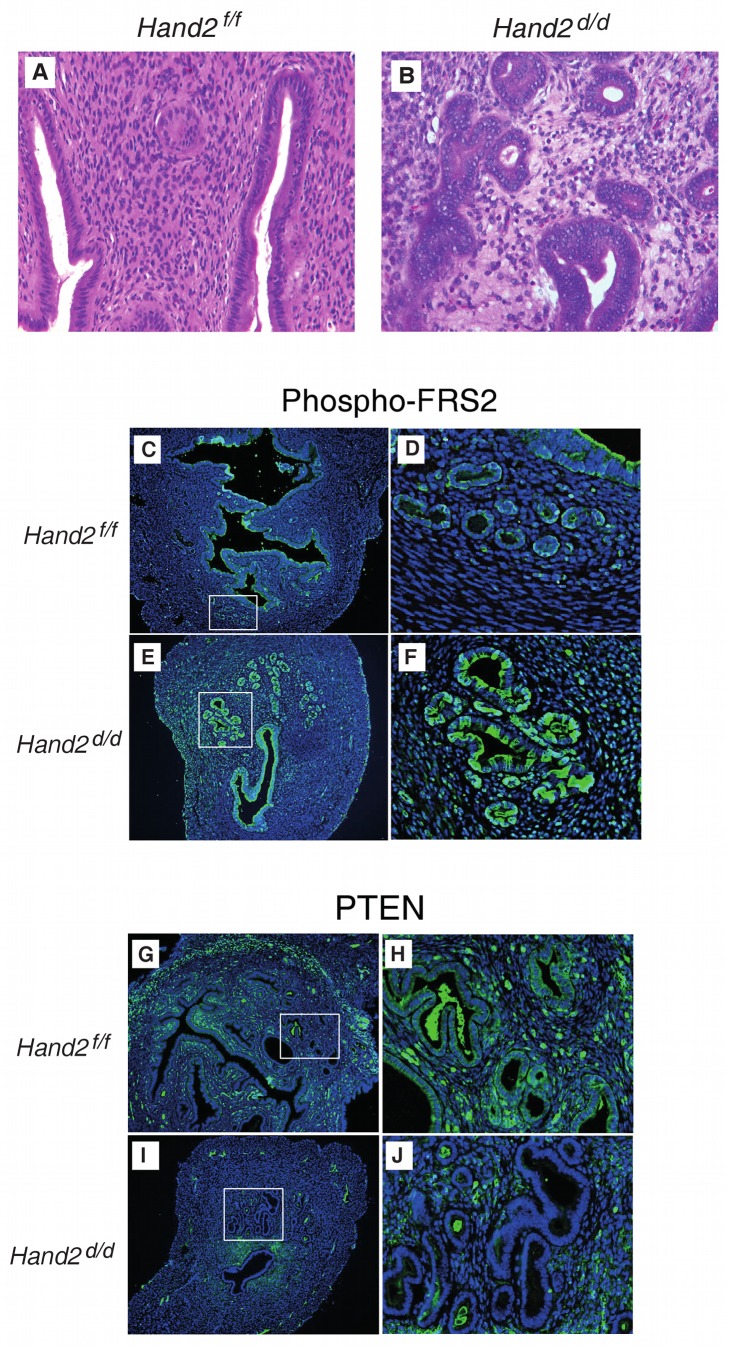
Endometrial pre-invasive neoplastic changes in *Hand2* conditional knock-out mice. (A and B) Hematoxylin and eosin staining of uterine sections from *Hand2^f/f^* (A) and *Hand2^d/d^* (B) mice (*n* = 5). Representative uterine sections obtained from mice at 24–48 wk of age are shown. Closely packed, irregular-shaped glands and features consistent with CAH are evident in uteri lacking *Hand2*. Note multiple layers of glandular epithelium in *Hand2^d/d^* uteri. Magnification 40×. (C–F) Phospho-FRS2 immunofluorescence in sections from *Hand2^f/f^* (C) (boxed region in [C] further magnified in [D]) and *Hand2^d/d^* (E) (boxed region in [E] further magnified in [F]) mice (*n* = 3). Magnification 20× (C and E) and 40× (D and F). (G–J) PTEN immunofluorescence in sections from *Hand2^f/f^* (G) (boxed region in [G] further magnified in [H]) and *Hand2^d/d^* (I) (boxed region in [I] further magnified in [J]) mice. Magnification 20× (G and I) and 40× (H and J) (*n* = 3).

In humans, age and a history of long-term progesterone/oestrogen imbalance (for which *HAND2* methylation may potentially be the resulting final molecular surrogate) are the major risk factors for endometrial cancer. Hence, to further investigate the functional role of *HAND2* silencing in the earliest stages of endometrial cancer development, we studied changes in endometrial histology as a function of age in mice with a conditional knock-out of *Hand2* in uterine tissue. As in a previous study [23], mice harbouring the floxed *Hand2* gene (*Hand2^f/f^*) were crossed with PR-Cre mice (in which Cre recombinase was inserted into the PR gene) in order to generate *Hand2^d/d^* mice in which the *Hand2* gene is deleted selectively in cells expressing PR (these mice express a fully functional PR [23]). Both *Hand2^d/d^* and *Hand2^f/f^* mice were healthy before tissue collection. No differences in appearance or body weight were observed between these two groups. Representative uterine sections obtained from *Hand2^f/f^* and *Hand2^d/d^* mice (*n* = 24) that were studied at 8–10, 24–32, and 40–48 wk of age are shown in Figure 4A–4F. We observed a significant increase in the gland/stroma ratio (Figure 4G) and an irregularity in the shape and size of the glands in *Hand2^d/d^* uteri compared to *Hand2^f/f^* uteri with increasing age (Figure 4A–4F). In addition, the endometrium of *Hand2^d/d^* mice demonstrated clear histological features of CAH (Figure 5A and 5B), including increased mitotic activity (Figure S11). This indicates that disruption of Hand2-mediated signalling solely in cells expressing the PR leads to morphological changes in the endometrium (i.e., CAH) associated with a very high likelihood of invasive endometrial cancer development [33].

### Conditional Knock-Out of *Hand2* in Uterine Tissue Leads to Molecular Changes Commonly Observed in Human Endometrial Cancer

In the uterine stroma Hand2 suppresses the production of several fibroblast growth factors (FGFs) that act as paracrine mediators of the mitogenic effects of oestrogen on the epithelium [23]. FGF receptor substrate 2 (FRS2) participates in the transmission of extracellular signals from the FGF receptor, and activation of the FGF receptor and phosphorylation of FRS2 [34] are a crucial event for the development of some cancers. *Hand2* conditional knock-out mice demonstrate substantially increased epithelial phospho-FRS2, indicating an increase of FGF receptor signalling in *Hand2*-null mice during uterine hyperplasia (Figure 5C–5F).

*PTEN* mutation and suppression has been shown to be one of the earliest and most frequently observed features in human endometrial carcinogenesis, and in mice, loss of *Pten* is sufficient to cause endometrial carcinogenesis [35]. Unlike control mice, *Hand2* conditional knock-out mice lack Pten expression in the endometrial epithelium (Figure 5G–5J), suggesting that *HAND2* silencing is a crucial step in endometrial carcinogenesis. In contrast, altered p53 expression is not an early event in human endometrial carcinogenesis [36], and our findings in mice are consistent with this observation (Figure S12).

## Discussion

Whereas there is little doubt that genetic alterations are required for cancer development, the causal role of the epigenome in this process is still under debate. Here we used a multifaceted approach to assess the role of DNAme in endometrial carcinogenesis. We began with a novel bioinformatics strategy—the EpiMod algorithm—to identify the top candidates most likely involved in endometrial carcinogenesis. We then sequenced the top-ranked gene—*HAND2*—and validated DNAme and RNA expression of this gene in an additional set of endometrial cancer and control tissues and, furthermore, compared *HAND2* methylation to other common molecular alterations in endometrial cancer. We studied *HAND2* methylation and protein expression at various stages of endometrial premalignant development and confirmed that methylation of this gene is an early event. We found that *HAND2* methylation is able to predict response to progesterone and provides a sensitive test to correctly identify endometrial cancer patients amongst those women who present with postmenopausal bleeding through the DNAme analysis of endometrial secretions on high vaginal swabs. Finally, we studied the effect of endometrial *Hand2* deletion in a mouse model and found that the absence of *Hand2* triggers pre-neoplastic alterations with increasing age.

We provide supporting evidence to suggest that PCGT methylation, as exemplified here by *HAND2* DNAme (the hub of the top-ranked EpiMod hotspot), is not merely a passive epigenetic feature of cancer but plays a functional role that facilitates the carcinogenic process—i.e., in the development of endometrial premalignancy (CAH).

*HAND2* exhibits all the features of a classical tumour suppressor gene: (1) it is activated by progesterone, which is considered to be the ultimate endometrial tumour suppressor [14]; (2) it suppresses oestrogen-mediated signals (e.g., FGFs) that stimulate the endometrial epithelium [23] and are known to be involved in endometrial carcinogenesis [37]; (3) it is robustly suppressed in endometrial cancer by means of a covalent modification (methylation) of DNA; (4) it is the hub of a differential methylation hotspot that ranked top among all hotspots in an integrative epigenome-interactome network analysis; (5) DNAme of *HAND2* increases with the development of endometrial premaligancy and is associated with resistance to progesterone; and (6) deletion of *Hand2* in mice leads to morphological as well as molecular changes that precede invasive endometrial cancer.

When compared to other frequent DNA-based alterations in endometrial cancers such as *p53*, *PTEN*, and *PIK3CA* mutations or microsatellite instability [38],[39], *HAND2* DNAme was found to be the most common DNA-based alteration. Applying a conservative threshold (the highest methylation level in normal endometrial samples), *HAND2* hypermethylation is present in >90% of all endometrioid endometrial cancers. Detection of *HAND2* DNAme could potentially afford multiple clinical utilities including risk prediction and early detection of endometrial cancer in women presenting with postmenopausal bleeding, as well as prediction of treatment response for confirmed disease.

Whilst it is currently technologically impossible to specifically hypermethylate and silence individual genes, we also show that mice exhibiting a knock-out of *Hand2* in progesterone-expressing endometrial cells develop abnormal endometrial histology with increasing age. Importantly, the observed lesions mimic CAH, which represents the initial stage of endometrial cancer development in humans and is associated with suppression of genes, including *PTEN*, that are known to be frequently mutated and suppressed in human endometrial cancers.

Our data have two major implications. First, we provide supporting evidence that suggests epigenetic aberrations, i.e., stem cell PCGT DNAme, are functionally important and contribute significantly to carcinogenesis and are not simply passive cancer characteristics. Although we and others have previously demonstrated that PCGT methylation is a hallmark of cancer [5],[21],[40]–[43], it has remained unclear whether methylation of these genes represents an epiphenomenon or has functional relevance in early carcinogenesis. We believe our research approach—i.e., (1) application of the FEM algorithm followed by (2) analysis of identified functional EpiMod hotspots in early stage premalignancy and malignancy in humans, and finally, (3) comparative assessment of results using conditional knock-out animal models—presents an analytical strategy that could be applied by others to discover those genes that are both epigenetically regulated and functionally important in the development of other cancers.

Second, endometrial cancer is the most common of all gynaecological cancers, and its incidence is continuing to rise dramatically owing to the current ageing and obesity epidemics. Consequently, novel strategies to prevent and/or early detect this disease are very much required. The potential clinical utility of *HAND2* DNAme analysis is significant in that it could be applied to triage women who present with postmenopausal bleeding (currently ∼90% of women who present with this symptom and are cancer-free must undergo endometrial biopsy for a definitive diagnosis) and could be further employed as a test to early detect endometrial cancer and predict response to preventative treatment.

Despite the notable findings and comprehensive nature of this study, we acknowledge that some study limitations remain: (1) the immediate consequences of *HAND2* silencing in endometrial stroma cells on both the molecular and cellular level require further assessment using laser-assisted micro-dissection and various primary cell culture assays; (2) suggested clinical applications of a *HAND2* methylation test, i.e., for the purposes of early detection and treatment prediction would require validation in both prospective settings and clinical trials; and (3) further studies need to be performed to address the role of epigenetic alterations in the less common non-endometrioid endometrial cancer subtypes including serous and clear cell endometrial cancers.

## Supporting Information

Figure S1 **DNA methylation level at specific CpGs of seven genes known to be hypermethylated** [**20**] **.** DNAme was analysed by means of the Illumina Infinium HumanMethylation27K array in 23 normal and 64 endometrial cancer samples (Set 1; Table S1). β-values for all CpGs for the seven genes indicated are blotted, and a Wilcoxon rank sum test *p*-value is provided. C, cancer; N, normal.
(TIF)

Figure S2 **Phospho-FSR2 and PTEN immunofluorescence negative controls.** Magnification 40× and 20×, respectively.
(TIF)

Figure S3 **The Functional Epigenetic Modules algorithm: integration of epigenome-transcriptome-interactome data to identify epigenetic drivers in cancer.** Step 1: Differential methylation statistics are overlaid onto a protein interaction network, and hotspots of differential methylation are inferred using a module detection algorithm as described in Methods (blue = hypermethylation in cancer, orange = hypomethylation in cancer). Step 2: Differential expression statistics are overlaid onto the same protein interaction network, and hotspots of simultaneous differential methylation and differential expression are inferred using the module detection algorithm on the integrated weighted network as described in Methods (red = overexpression in cancer, green = underexpression in cancer).
(TIF)

Figure S4 **Principal component analysis in Set 1.** Left panel is a scatterplot of the weights in the top two principal component analysis components. Right panel is a boxplot of the weights in the top singular principal component analysis component. Wilcoxon rank sum test *p*-value for a difference between the weights in normal and cancer tissue is given.
(TIF)

Figure S5 ***HAND2*** **mRNA expression in normal and cancerous endometrium (Set 2).** Wilcoxon rank sum test *p*-value is given. EC, endometrial cancer; N, normal.
(TIF)

Figure S6 **Heatmap of Illumina Infinium HumanMethylation27K DNA methylation levels (Set 1) of significantly hypermethylated** ***HAND2*** **epigenetic module members.**
(TIF)

Figure S7 **Scatterplots of** ***HAND2*** **versus** ***DCC*** **DNA methylation and mRNA expression.** Left panel: Scatterplot of *HAND2* and *DCC* DNAme levels (Set 1). Right panel: Scatterplot of *HAND2* and *DCC* mRNA expression levels (Set 2). Green and red indicate normal and cancer, respectively.
(TIF)

Figure S8 **Correlation between two differentially located CpG sites analysed using the Illumina Infinium HumanMethylation27K bead array, and two differentially located MethyLight reactions (designed to cover the Illumina CpG sites) for the** ***HAND2*** **gene.** Left panel: cg02774439 represents a CpG site located +127 bp downstream of the transcription start site within the CpG island in the 5′ untranslated region, and cg01580681 is located +1,362 bp downstream of the transcription start site within exon 1. Right panel: The MethyLight reaction ML_HAND2_I (incorporating cg02774439)—a 83-bp real-time PCR reaction beginning +51 downstream of the transcription start site within the CpG island in the 5′ untranslated region—was compared with ML_HAND2_II (incorporating cg01580681)—a 78-bp real-time PCR reaction beginning +1,355 downstream of the transcription start site within exon 1. Refer to Figure 1E for a schematic of the CpG locations within the *HAND2* gene.
(TIF)

Figure S9 **Association between sequence variations in the 5′ region of** ***HAND2*** **and DNA methylation.** cg02774439 represents a CpG site located +127 bp downstream of the transcription start site within the CpG island in the 5′ untranslated region, and cg01580681 is located +1,362 bp downstream of the transcription start site within exon 1. In 23 endometrial cancer samples, the entire region +1 transcription start site to +2,071 bp downstream of the transcription start site was sequenced (Table S5), and *HAND2* DNAme levels were plotted for samples with sequence variants absent or present in the 2,070-bp region.
(TIF)

Figure S10 **Sensitivity and specificity of vaginal swab** ***HAND2*** **methylation to diagnose stage greater than stage 1A endometrial cancer.** ROC curves measuring the sensitivity and specificity of *HAND2* methylation in vaginal swabs to discriminate women with a greater than stage 1A endometrial cancer (*n* = 13) from women with non-cancerous causes (*n* = 17) for postmenopausal bleeding. AUC and *p*-values (P) as specified (see also Table S3).
(TIF)

Figure S11 **Increased mitotic activity in** ***Hand2^d/d^*** **knock-out mice versus controls.** Uterine sections from *Hand2^f/f^* (A) and *Hand2^d/d^* (B) mice (*n* = 5) were subjected to immunohistochemical staining with Ki67, a marker of cell proliferation. Note the hyperproliferative glandular epithelium in uteri lacking *Hand2*. L, G, and S indicate lumen, glands, and stroma, respectively. The measurement of glandular epithelial cell proliferation in uterine sections of *Hand2^f/f^* and *Hand2^d/d^* mice was performed by immunostaining for Ki67. Digital images of immunostained sections of uteri from *Hand2^f/f^* and *Hand2^d/d^* mice (*n* = 5) were captured and analysed. Quantification of Ki67-positive cells was performed using Image J software (http://rsb.info.nih.gov/ij/) with cell counter plug-in. For each sample, the Ki67-positive cells and total number of cells per field were counted for an average of 8–10 fields per section, and the average percentage positive cells was calculated. Data are expressed as mean ± standard error of the mean, and comparisons between experimental groups are made (C) using analysis of variance. Statistical significance was assigned at *p*<0.05.
(TIF)

Figure S12 **P53 immunofluorescence in** ***Hand2^d/d^*** **knock-out mice versus controls.** (A–D) Uterine sections from *Hand2^f/f^* (A and C) (*n* = 3) and *Hand2^d/d^* mice (B and D) (*n* = 3) were subjected to immunofluorescence staining with p53 antibody. Magnification 20× (A and B) and 40× (C and D). (E) negative control. Note there is no difference in p53 staining between *Hand2^f/f^* and *Hand2^d/d^* mice.
(TIF)

Table S1 **Associations of** ***HAND2*** **DNAme and clinicopathological data for sample Set 1.**
(XLS)

Table S2 ***HAND2*** **methylation (MethyLight reaction ML_HAND2_II in** **Figure 1E** **) in Set 3.**
(XLS)

Table S3 ***HAND2*** **methylation in vaginal swabs from women with postmenopausal bleeding (Set 4).**
(XLS)

Table S4 **Clinical information for patients undergoing treatment with progesterone (Set 6).**
(XLS)

Table S5 ***HAND2*** **sequencing results (Sanger sequencing) in Set 1.**
(XLS)

Table S6 **Ranked list of genes hypermethylated in cancerous compared to normal endometrium.**
(XLS)

Table S7 **Gene Set Enrichment Analysis of the top hypermethylated genes in endometrial cancer.**
(XLS)

Table S8 **Gene Set Enrichment Analysis of the top hypomethylated genes in endometrial cancer.**
(XLS)

Table S9 **Ranked list of top 19 epigenetic modules from the epigenome-interactome analysis.**
(XLS)

Table S10 **Detailed compositions of the top 19 epigenetic modules.**
(XLS)

Table S11 **Gene Set Enrichment Analysis of those genes comprising each of the 19 top-ranked epigenetic modules.**
(XLS)

Table S12 ***HAND2*** **methylation (Illumina Infinium HumanMethylation27K array data) in Set 1 and detailed associated molecular characteristics of the individual tumours.**
(XLS)

Text S1 **Supplementary methods.**
(DOCX)

Text S2 **ARRIVE document.**
(DOC)

## Abbreviations

AUC: area under the receiver operating characteristic curve
CAH: complex atypical hyperplasia
DNAme: DNA methylation
EpiMod: epigenetic module
FEM: Functional Epigenetic Modules
FGF: fibroblast growth factor
GSEA: Gene Set Enrichment Analysis
PCGT: polycomb group target gene
PMR: percentage of methylated reference
PR: progesterone receptor
ROC: receiver operating characteristic
