# Supplementary figures and images for: Role of DNA Methylation and Epigenetic Silencing of HAND2 in Endometrial Cancer Development
Source: PLoS Med. 2013 Nov 12;10(11):e1001551. doi: 10.1371/journal.pmed.1001551 (PMC3825654; doi:10.1371/journal.pmed.1001551)

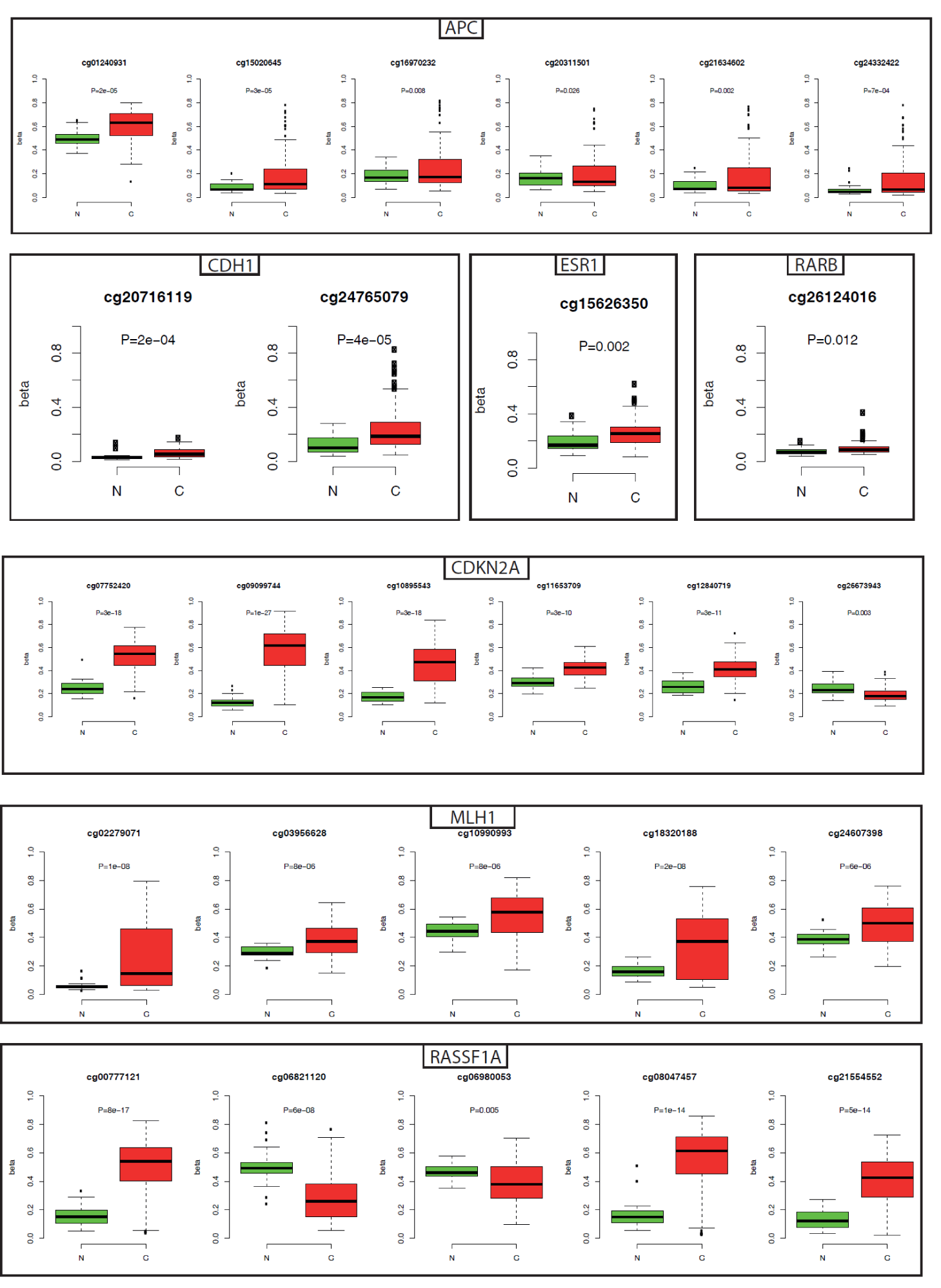

Supplement: Figure S1 — DNA methylation level at specific CpGs of seven genes known to be hypermethylated [20] . DNAme was analysed by means of the Illumina Infinium HumanMethylation27K array in 23 normal and 64 endometrial cancer samples (Set 1; Table S1). β-values for all CpGs for the seven genes indicated are blotted, and a Wilcoxon rank sum test p-value is provided. C, cancer; N, normal. (TIF) [file pmed.1001551.s001.tif]

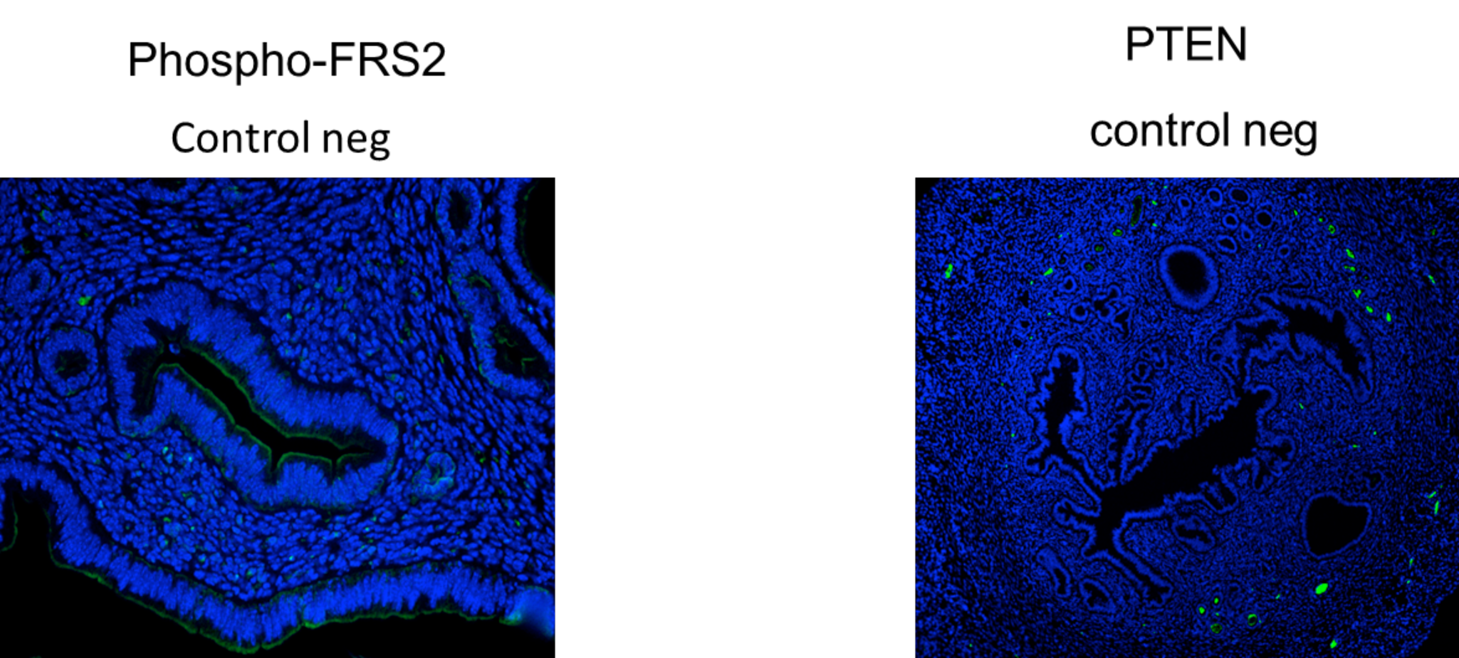

Supplement: Figure S2 — Phospho-FSR2 and PTEN immunofluorescence negative controls. Magnification 40× and 20×, respectively. (TIF) [file pmed.1001551.s002.tif]

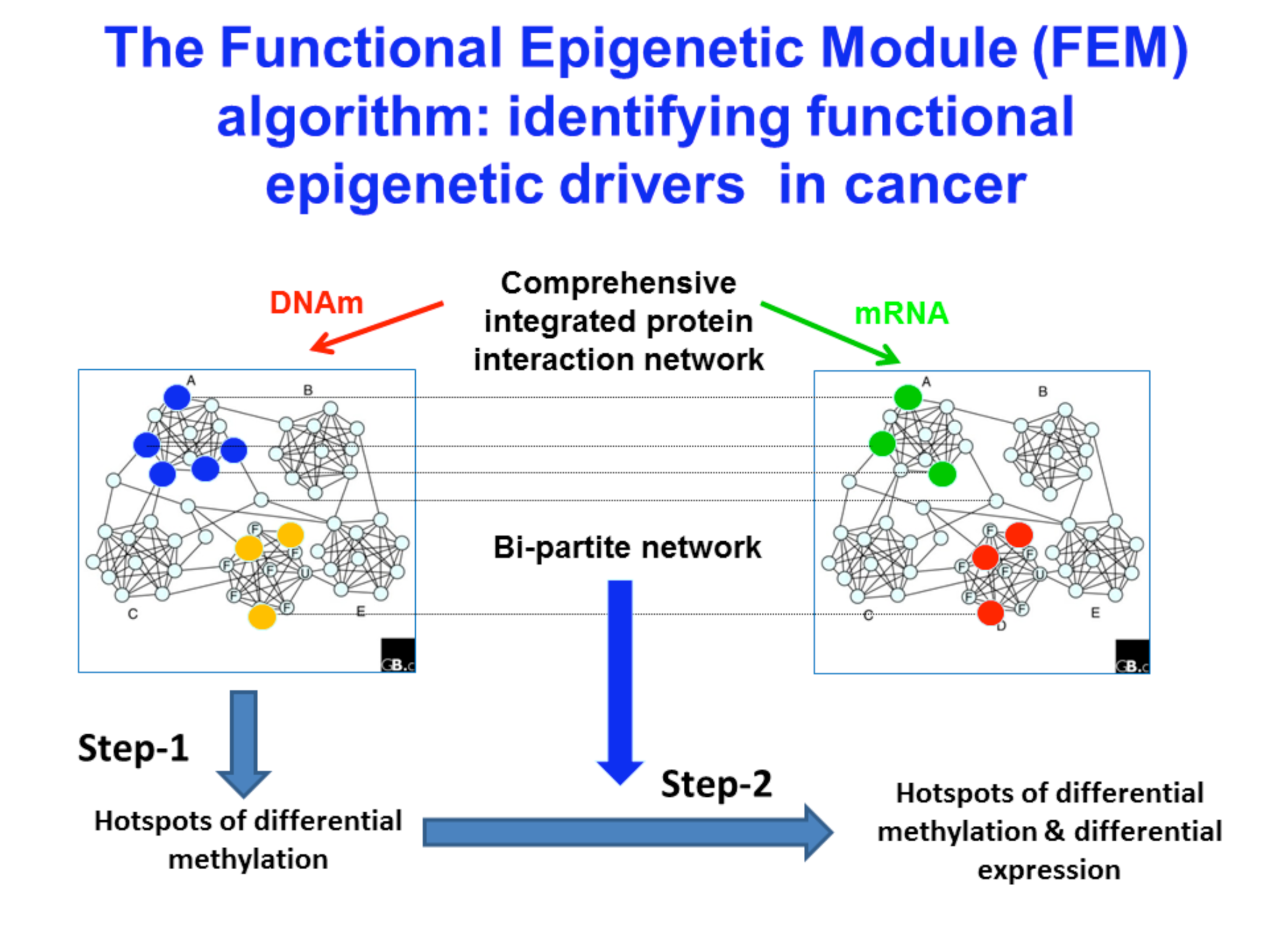

Supplement: Figure S3 — The Functional Epigenetic Modules algorithm: integration of epigenome-transcriptome-interactome data to identify epigenetic drivers in cancer. Step 1: Differential methylation statistics are overlaid onto a protein interaction network, and hotspots of differential methylation are inferred using a module detection algorithm as described in Methods (blue = hypermethylation in cancer, orange = hypomethylation in cancer). Step 2: Differential expression statistics are overlaid onto the same protein interaction network, and hotspots of simultaneous differential methylation and differential expression are inferred using the module detection algorithm on the integrated weighted network as described in Methods (red = overexpression in cancer, green = underexpression in cancer). (TIF) [file pmed.1001551.s003.tif]

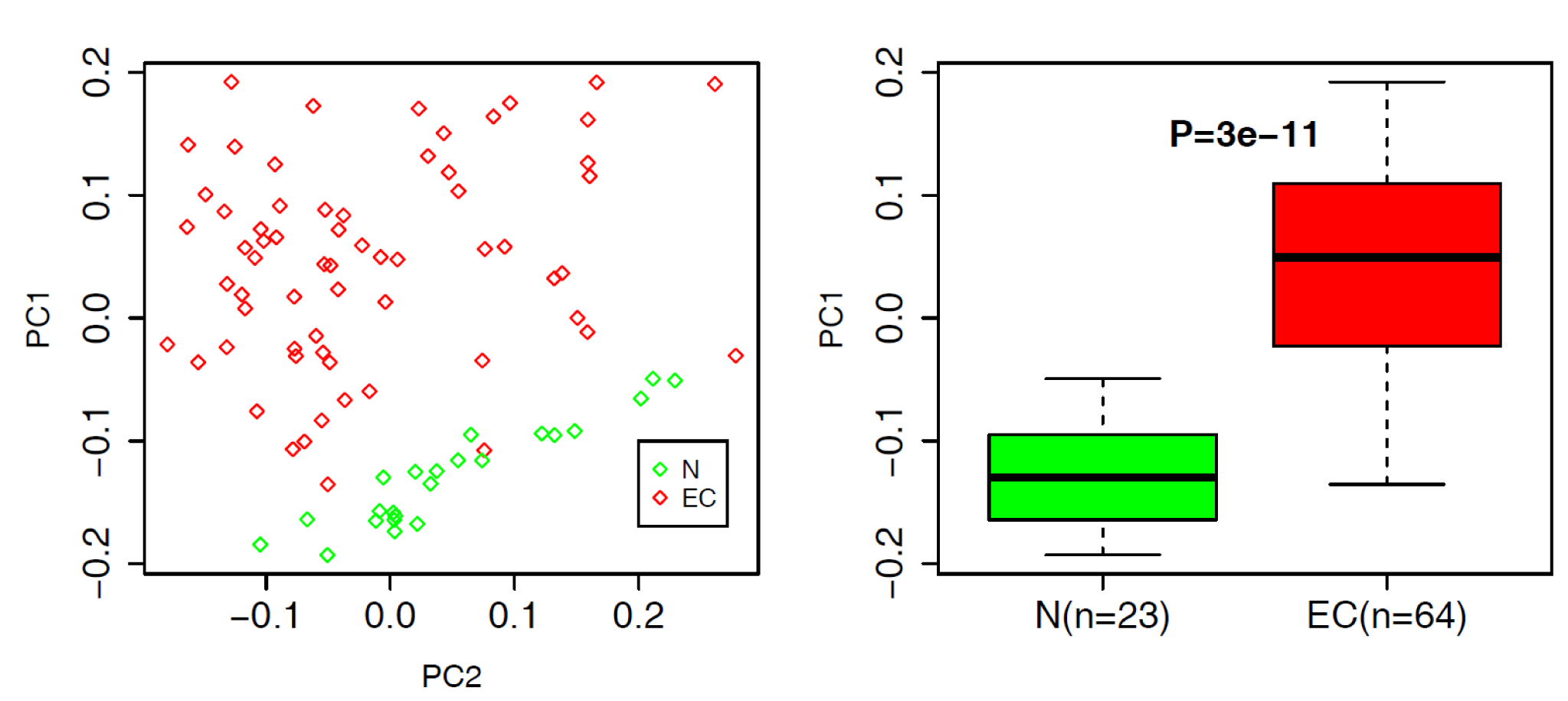

Supplement: Figure S4 — Principal component analysis in Set 1. Left panel is a scatterplot of the weights in the top two principal component analysis components. Right panel is a boxplot of the weights in the top singular principal component analysis component. Wilcoxon rank sum test p-value for a difference between the weights in normal and cancer tissue is given. (TIF) [file pmed.1001551.s004.tif]

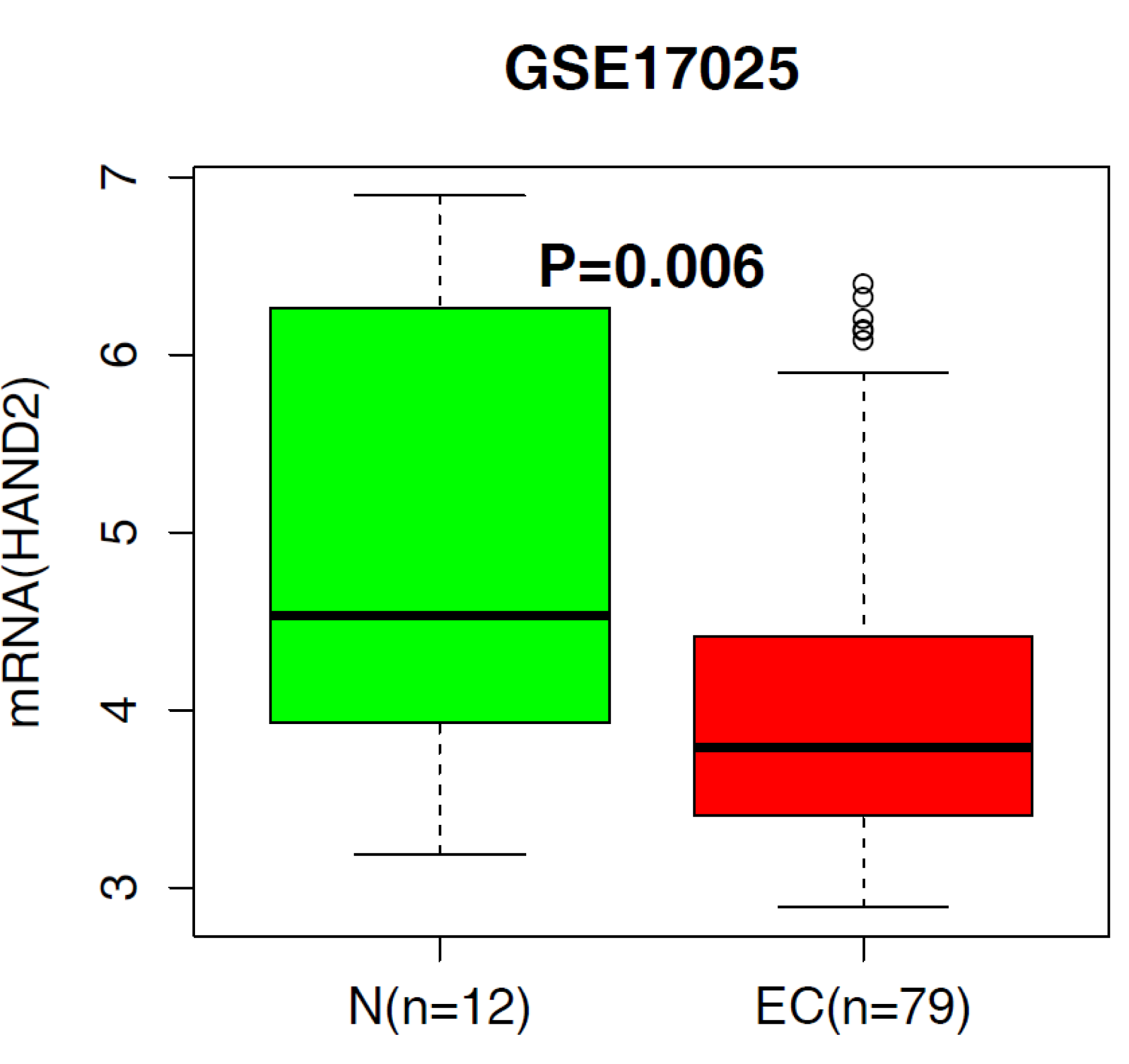

Supplement: Figure S5 — HAND2 mRNA expression in normal and cancerous endometrium (Set 2). Wilcoxon rank sum test p-value is given. EC, endometrial cancer; N, normal. (TIF) [file pmed.1001551.s005.tif]

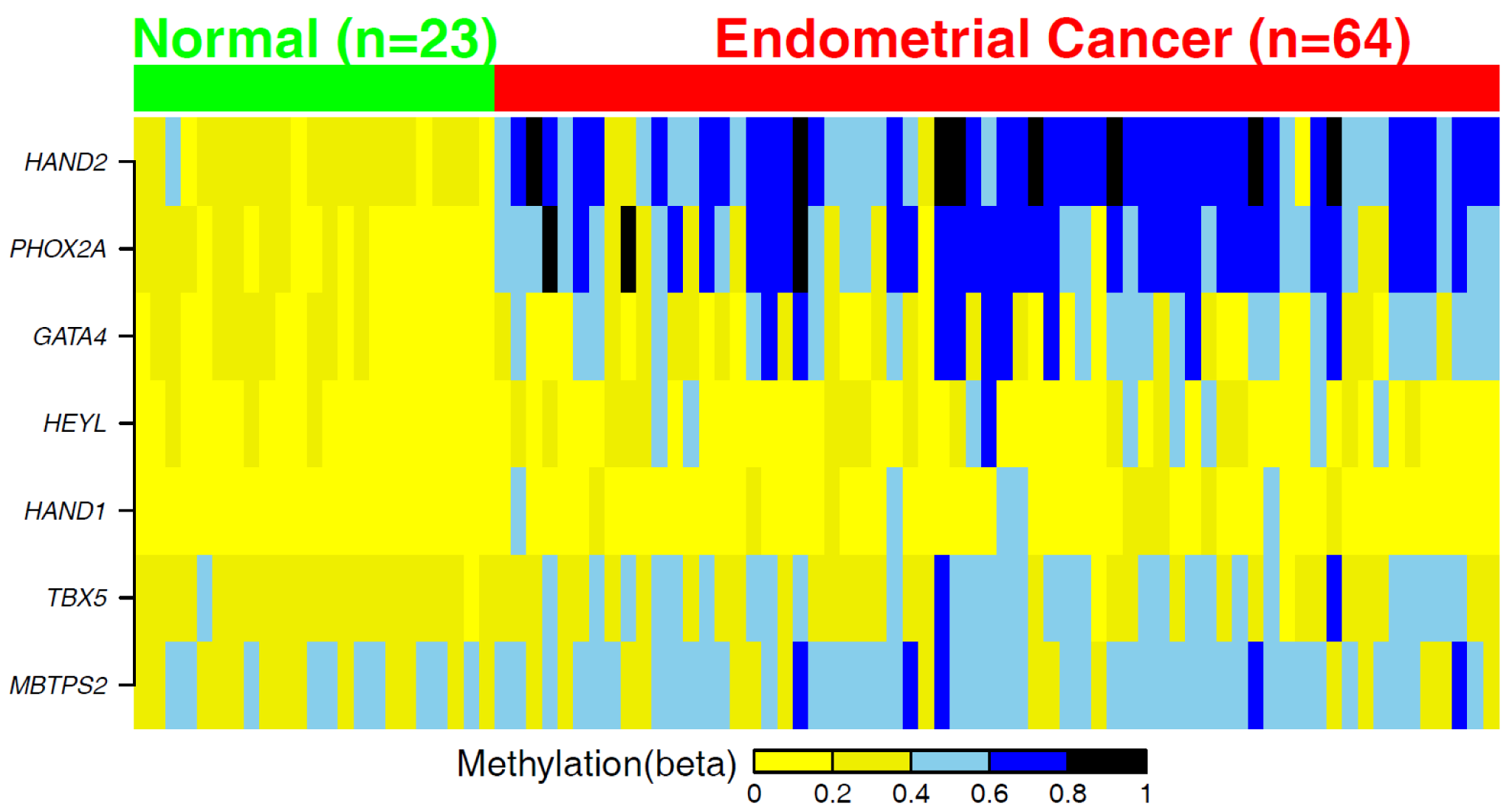

Supplement: Figure S6 — Heatmap of Illumina Infinium HumanMethylation27K DNA methylation levels (Set 1) of significantly hypermethylated HAND2 epigenetic module members. (TIF) [file pmed.1001551.s006.tif]

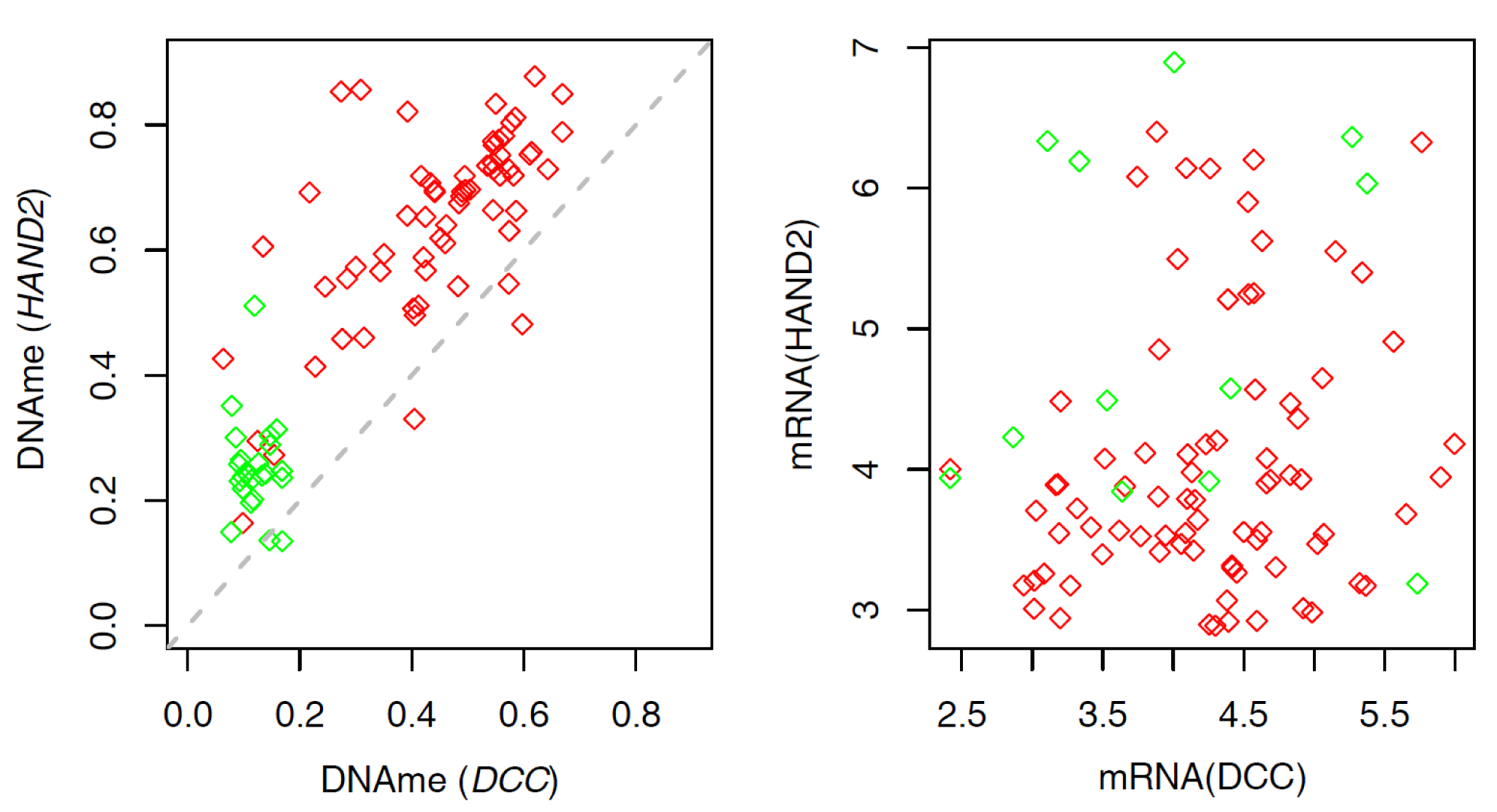

Supplement: Figure S7 — Scatterplots of HAND2 versus DCC DNA methylation and mRNA expression. Left panel: Scatterplot of HAND2 and DCC DNAme levels (Set 1). Right panel: Scatterplot of HAND2 and DCC mRNA expression levels (Set 2). Green and red indicate normal and cancer, respectively. (TIF) [file pmed.1001551.s007.tif]

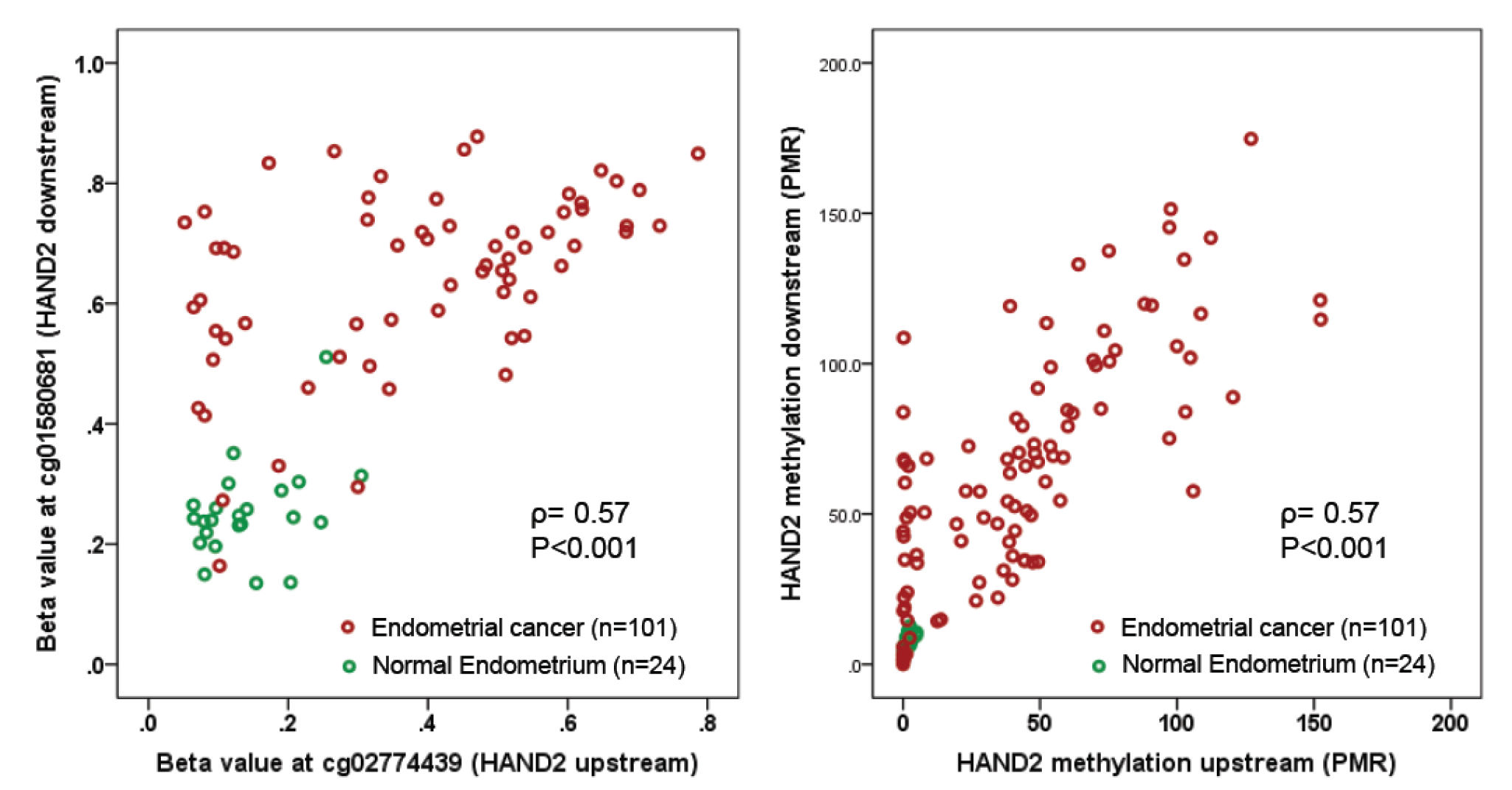

Supplement: Figure S8 — Correlation between two differentially located CpG sites analysed using the Illumina Infinium HumanMethylation27K bead array, and two differentially located MethyLight reactions (designed to cover the Illumina CpG sites) for the HAND2 gene. Left panel: cg02774439 represents a CpG site located +127 bp downstream of the transcription start site within the CpG island in the 5′ untranslated region, and cg01580681 is located +1,362 bp downstream of the transcription start site within exon 1. Right panel: The MethyLight reaction ML_HAND2_I (incorporating cg02774439)—a 83-bp real-time PCR reaction beginning +51 downstream of the transcription start site within the CpG island in the 5′ untranslated region—was compared with ML_HAND2_II (incorporating cg01580681)—a 78-bp real-time PCR reaction beginning +1,355 downstream of the transcription start site within exon 1. Refer to Figure 1E for a schematic of the CpG locations within the HAND2 gene. (TIF) [file pmed.1001551.s008.tif]

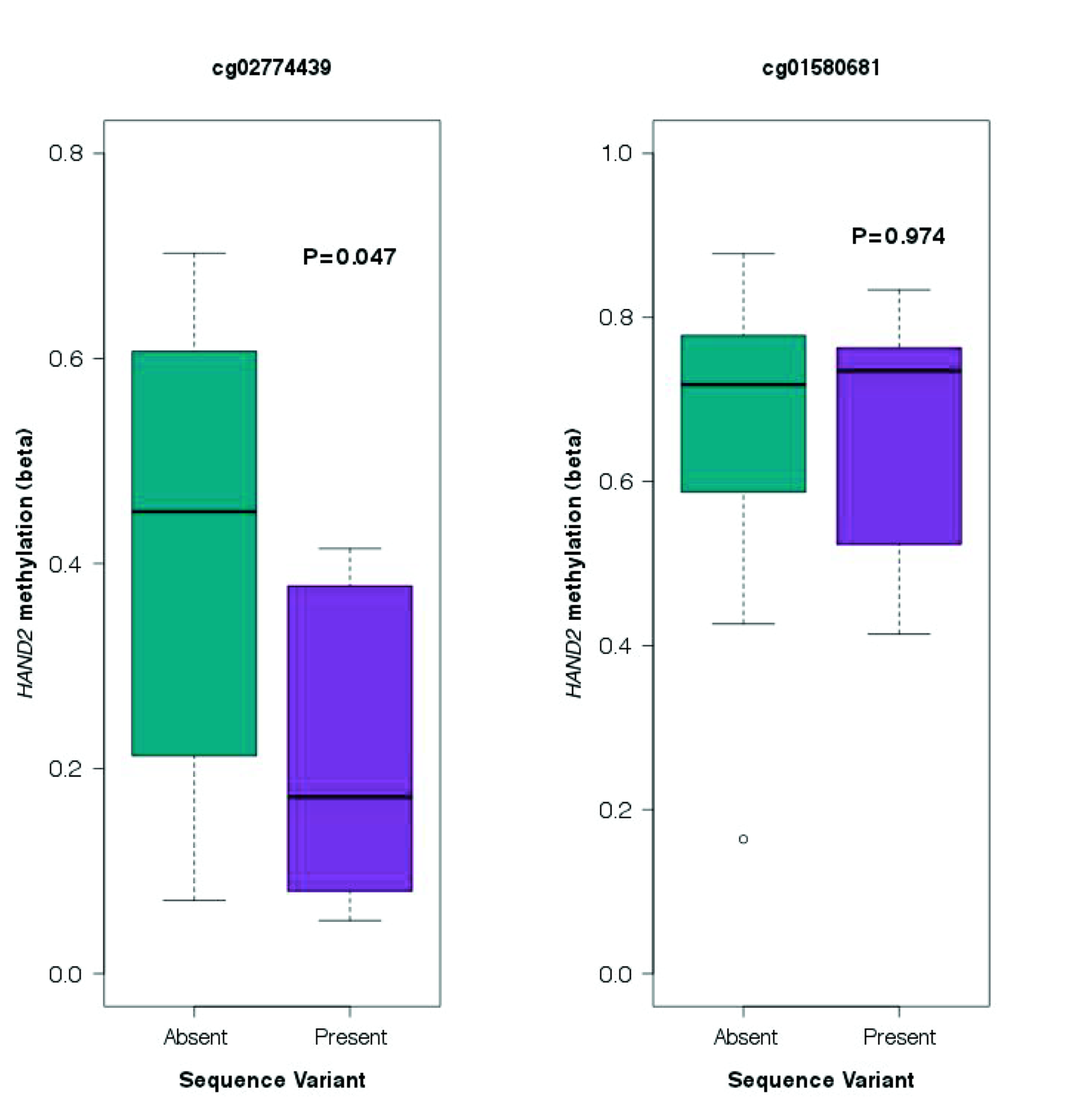

Supplement: Figure S9 — Association between sequence variations in the 5′ region of HAND2 and DNA methylation. cg02774439 represents a CpG site located +127 bp downstream of the transcription start site within the CpG island in the 5′ untranslated region, and cg01580681 is located +1,362 bp downstream of the transcription start site within exon 1. In 23 endometrial cancer samples, the entire region +1 transcription start site to +2,071 bp downstream of the transcription start site was sequenced (Table S5), and HAND2 DNAme levels were plotted for samples with sequence variants absent or present in the 2,070-bp region. (TIF) [file pmed.1001551.s009.tif]

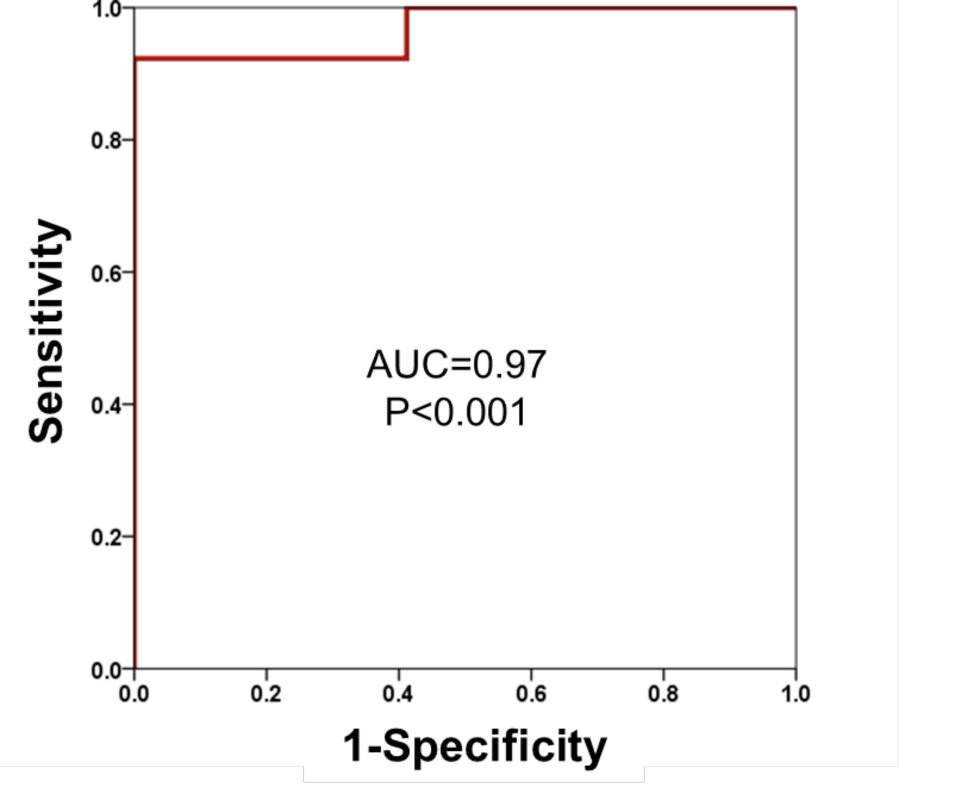

Supplement: Figure S10 — Sensitivity and specificity of vaginal swab HAND2 methylation to diagnose stage greater than stage 1A endometrial cancer. ROC curves measuring the sensitivity and specificity of HAND2 methylation in vaginal swabs to discriminate women with a greater than stage 1A endometrial cancer (n = 13) from women with non-cancerous causes (n = 17) for postmenopausal bleeding. AUC and p-values (P) as specified (see also Table S3). (TIF) [file pmed.1001551.s010.tif]

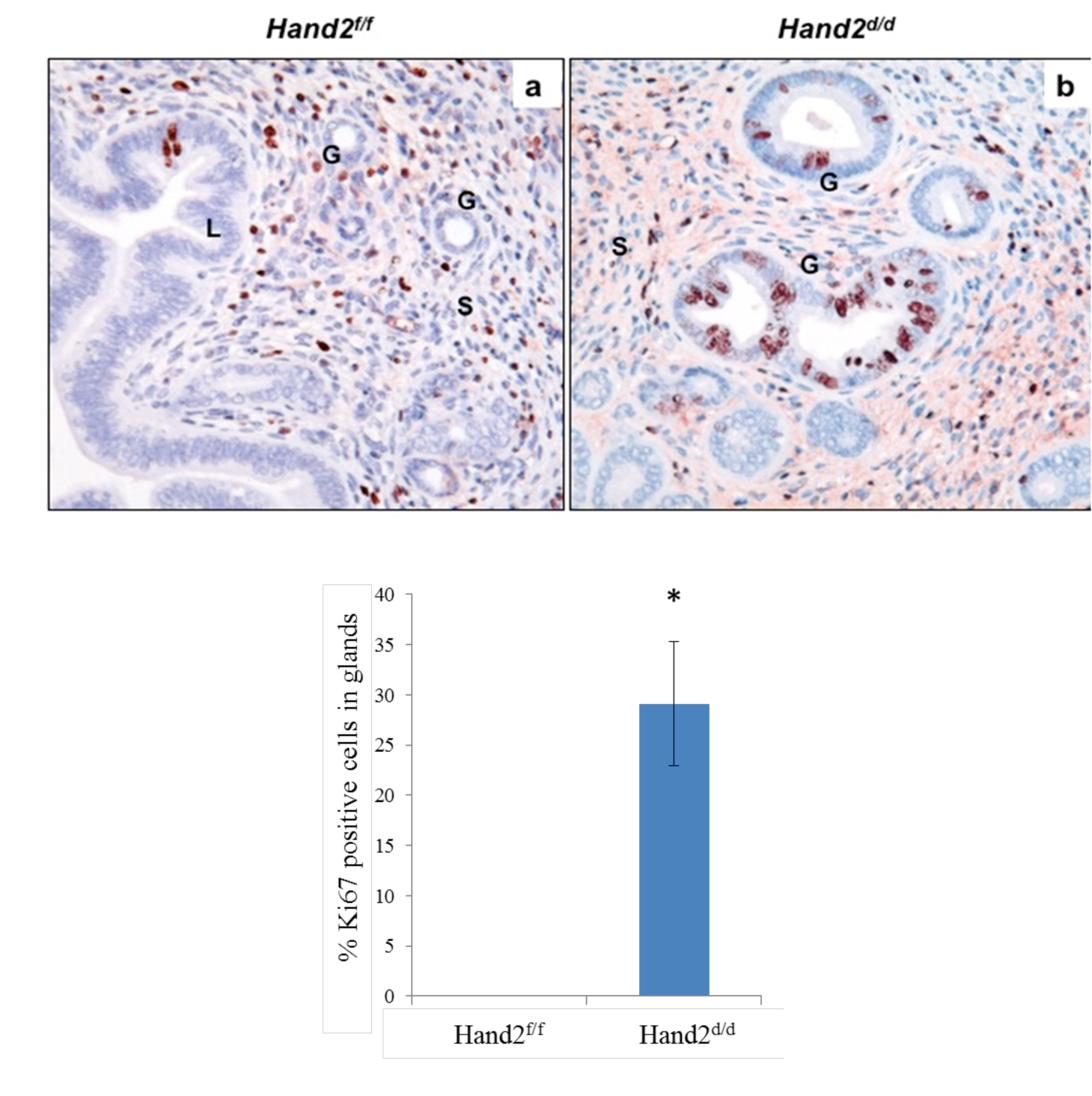

Supplement: Figure S11 — Increased mitotic activity in Hand2d/d knock-out mice versus controls. Uterine sections from Hand2f/f (A) and Hand2d/d (B) mice (n = 5) were subjected to immunohistochemical staining with Ki67, a marker of cell proliferation. Note the hyperproliferative glandular epithelium in uteri lacking Hand2. L, G, and S indicate lumen, glands, and stroma, respectively. The measurement of glandular epithelial cell proliferation in uterine sections of Hand2f/f and Hand2d/d mice was performed by immunostaining for Ki67. Digital images of immunostained sections of uteri from Hand2f/f and Hand2d/d mice (n = 5) were captured and analysed. Quantification of Ki67-positive cells was performed using Image J software (http://rsb.info.nih.gov/ij/) with cell counter plug-in. For each sample, the Ki67-positive cells and total number of cells per field were counted for an average of 8–10 fields per section, and the average percentage positive cells was calculated. Data are expressed as mean ± standard error of the mean, and comparisons between experimental groups are made (C) using analysis of variance. Statistical significance was assigned at p<0.05. (TIF) [file pmed.1001551.s011.tif]

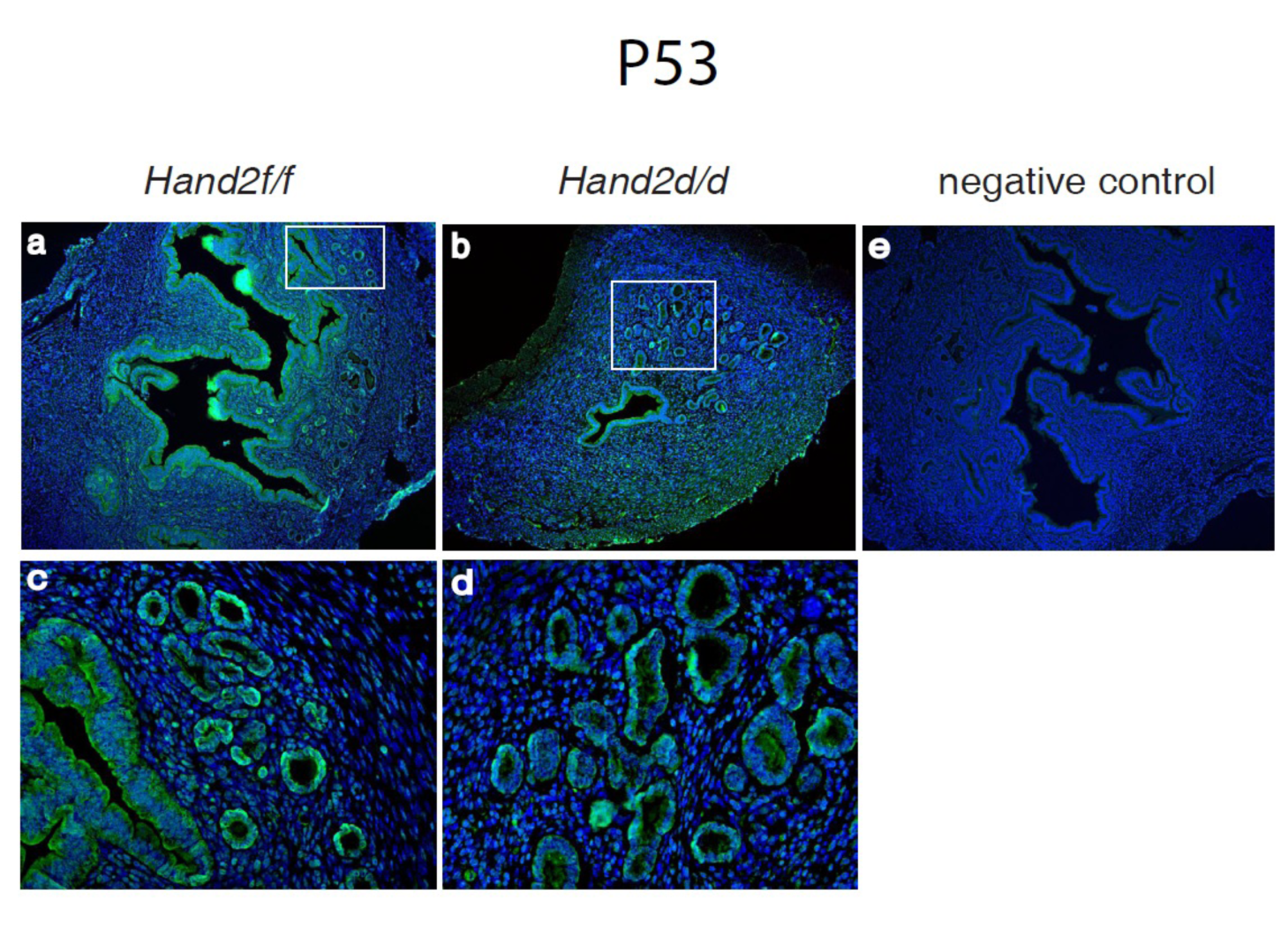

Supplement: Figure S12 — P53 immunofluorescence in Hand2d/d knock-out mice versus controls. (A–D) Uterine sections from Hand2f/f (A and C) (n = 3) and Hand2d/d mice (B and D) (n = 3) were subjected to immunofluorescence staining with p53 antibody. Magnification 20× (A and B) and 40× (C and D). (E) negative control. Note there is no difference in p53 staining between Hand2f/f and Hand2d/d mice. (TIF) [file pmed.1001551.s012.tif]
