# Supplementary material for: Role of DNA Methylation and Epigenetic Silencing of HAND2 in Endometrial Cancer Development
Source: PLoS Med. 2013 Nov 12;10(11):e1001551. doi: 10.1371/journal.pmed.1001551 (PMC3825654; doi:10.1371/journal.pmed.1001551)
Supplement: Text S1 — Supplementary methods. (DOCX) [file pmed.1001551.s025.docx]

METHODS (The EpiMod and Functional EpiMod (FEM) Algorithms)

**FUNCTIONAL EPIGENETIC MODULE ANALYSIS (The FEM Algorithm)**

**Synopsis:** The FEM algorithm uses an integrative epigenome-transcriptome-interactome approach to identify functionally and epigenetically deregulated interactome hotspots associated with a phenotype of interest, here endometrial cancer. There are 2 main steps to the algorithm (**see figure S3**): The first step is the EpiMod algorithm, and involves integration of the DNA methylation levels of gene promoters with a human interactome to identify differential methylation hotspots associated with endometrial cancer. In the second step, further integration with mRNA expression data is performed to identify epigenetically deregulated hotspots which may be of functional significance.

**Availability:** We have implemented the EpiMod and FEM algorithms as executable R-scripts, freely available from *http://code.google.com/p/epimods/*.

**The protein interaction network:** We downloaded an integrated protein interactome, including the Human Protein Reference Database (HPRD), from Pathway Commons *http://www.pathwaycommons.org* (*S1*) (13th June 2012). The network used here consisted of approximately 10,000 proteins with over 200,000 documented interactions. When restricted to the genes annotated on the Infinium 27k array, the resulting networks contained typically on the order of ~7500 nodes (proteins) and ~240,000 interactions (edges).

**Integration and edge weighting scheme:** First, we describe the EpiMod algorithm in the context of integrating DNA methylation data with a human interactome network, as used by us previously in the context of ageing (*S1*). Later we describe the novel integration to include also mRNA expression data (FEM). We assigned to each gene (protein) in the interaction network the methylation profile of the CpG closest to the transcription start site of that gene. For each node (gene) in the network we could thus assign a statistic (*S10*), *t_g_* , measuring the association between the methylation profile and endometrial cancer. Using these node statistics we could then assign to each edge in the network a weight, *w_gh_ ,*  between every pair of genes *g* and *h* in the underlying protein interaction network (PIN):

*w_gh_* = (|*t_g_*|+ |*t_h_|*)/(2|*t_max_*_|_)

where *t_max_*  is the maximum absolute statistic assigned to any gene. Note that if *g* and *h* do not interact we set *w_gh_=*0. This yields a weighting of each edge between 0 and 1, with larger weights denoting two neighbours both significantly associated with endometrial cancer.

**Validation of integration:** It is important to demonstrate that the integration of DNA methylation data with the protein interaction network results in an integrated network which contains biological information. This was demonstrated in West J et al Sci Rep 2013 (*S1*), where we showed that neighbours in the network exhibit stronger correlations in DNA methylation (as assessed over normal tissue samples only) than non-neighbours, a result which has also been shown to be valid in the context of gene expression data (*S2*).

**Identifying hotspots of alterations using a greedy spin-glass algorithm:** With the choice of edge weighting regime above, the task is to search the network for subnetworks of exceptionally large average weight density (“modules”' or “hotspots”) in relation to the network as a whole. The average weight density of a subnetwork we call modularity (*S3*). In order to identify the hotspots we adopted a greedy spin-glass algorithm from (*S4*), which allows community detection in weighted networks specified by a weighted adjacency matrix *W*. The justification for using this algorithm is as follows. First, we sought an algorithm that would allow flexibility in the level of mutual exclusivity of the inferred modules. Since proteins often lie in many distinct functional pathways, the algorithm must allow for some overlap between the inferred modules. On the other hand, the algorithm must also avoid inferring modules of very high overlap since this represents unwanted redundancy. One of the attractive features of the spin-glass algorithm is that it is characterized by only 1 tunable parameter, called γ with 0 ≤ γ ≤ 1, which, directly controls the average size and hence overlap/redundancy of the inferred modules. Another appealing feature of the spin-glass algorithm is that it allows for a greedy approach by starting the search of modules from seed nodes, defined as the nodes with the largest statistics. The top 100 ranked genes (CpGs), ranked by largest absolute statistic were declared as seeds. These all passed an FDR correction threshold of 0.05 based on their univariate P-values. It is important to point out however that not all seeds lead to modules of higher-than-average modularity, since some seeds may represent isolated nodes of association.

The spin-glass algorithm reduces the problem of community/module detection into finding the energy ground state of an infinite ranged Potts spin glass (*S4*). For each seed we thus obtain a module minimising an energy function using a simulated annealing procedure as implemented in the *spin-glass.community* function of the *igraph* R package. The minimisation is measured relative to null models consisting of random graphs with the same vertex degrees, amounting to a rewiring of the network. We point out again that the existence of a module associated with a given seed is not automatic since growing a module from a given seed may not lead to reductions in the overall energy function. Typically, one finds that approximately 50% of seeds are not associated with any module.

**Statistical significance of modules:** The statistical significance of the modules is further assessed using a permutation approach. Briefly, we compare the observed modularity value with the ones obtained by permuting the absolute t-statistics among nodes. By performing 1000 permutations, we can thus assess the statistical significance of the observed modularity values by counting the fraction of permutations which lead to higher modularities. We note that the modules inferred previously, i.e from the unpermuted node statistics, are kept fixed in this procedure and are not inferred de novo. In other words, only the assigned node attributes (i.e the absolute t-statistics) change between permutations, thus yielding an adjusted P-value for each separate module. We refer to the resulting modules which are significant at the 5% level, as EpiMods/Hotspots.

**Tuning the parameter γ:** We used the same γ value (=0.5) as in our previous study (*S1*). Briefly, this value typically yields modules in the size range 10 to 100 genes. As explained in West et al, there are two justifications for focusing on this size range. First, unsupervised linear decomposition methods like PCA or ICA performed on gene expression data (see e.g (*S5*)) suggest that co-regulated gene modules are typically on the order of 1% of the number of genes measured. In our network of ~7500 nodes this amounts to ~75 genes. A second more compelling justification was provided by a detailed analysis of the module size distribution, the mutual exclusivity of the resulting modules and an overall GSEA enrichment metric. This strategy allowed us to gauge the trade-off between the uniqueness of the modules (favouring smaller modules) and biological interpretation (favouring larger modules and requiring modules to be typically of size ≥10). We found that γ = 0.5 was optimal in the sense that it generated a reasonable number of gene modules of size ≥10, while also exhibiting minimal overlap. In comparison, γ = 1 yielded much larger, overlapping modules, leading to significant redundancy in downstream GSEA analyses. Importantly, we found that the optimal value (γ = 0.5) was very robust to the studies or phenotypes considered (*S1*). We point out again that the tuning of the parameter as described above was performed on independent data, analysis presented in West et al. *(S1*).

**Integrative epigenome-transcriptome-interactome analysis (Functional Epigenetic Module (FEM) Analysis):** We now describe the extension of the EpiMod algorithm to include transcriptomic data. In this case, we wish to assess if the differential methylation hotspots identified above remain significant hotspots in the context of both differential methylation and differential expression, whereby we require that the differential methylation near the TSS is anti-correlated with the differential expression change. In addition, the integrative analysis must allow for DNA methylation and gene expression data from independent (i.e. unmatched) samples. In our case, we are integrating the endometrial normal/cancer data from Sets 1 (DNA methylation) and 2 (mRNA expression), which are unmatched independent studies. In the integrated analysis, each node (gene) *i* in the network is associated with two attributes: a t-statistic of differential methylation *t_i_^(D)^* and a t-statistic of differential expression *t_i_^(R)^* . We note that the distribution of t-statistics from both assays were scaled so that the variance in statistics was the same from both assays. This was done to avoid bias favouring one particular data type in the inference procedure. We stress that the analysis was conducted using t-statistics (t-tests) and not logistic regressions since we wanted to use the same statistical test for both data types and t-test based statistics are the standard choice in differential expression analysis. Weights in the interactome between genes *i* and *j* were then defined as

*w_ij_ ~ H(t_i_^(D)^)H(-t_i_^(R)^)| t_i_^(D)^- t_i_^(R)^| + H(t_j_^(D)^)H(-t_j_^(R)^)| t_j_^(D)^- t_j_^(R)^|*

where *H(t)* is defined by *H(t)=*1 if *t* > 0, and *H(t)* =0 if *t <* 0. Thus, the above construction of the weights seeks hotspots where there is hypermethylation (*t^(D)^ >* 0) and corresponding underexpression ( *t^(R)^ <* 0) in cancer. Similarly, the definition of the weights can be easily modified to include the search for hypomethylated/overexpressed hotspots.

Seeds are defined as the top ranked genes under a combined statistic

*t_i_^(C)^ = H(t_i_^(D)^)H(-t_i_^(R)^)|t_i_^(D)^- t_i_^(R)^|*

and hotspots of coordinated hypermethylation/underexpression are subsequently inferred using the same procedure as described above. Statistical significance of resulting hotspots is assessed using the same randomisation procedure as described earlier.

**Supplementary References:**

S1. J. West , S. Beck, X. Wang, A.E. Teschendorff, Sci. Rep. **3**, 1630 (2013)

S2. I. W. Taylor *et al.*, *Nat. Biotechnol.* **27**, 199 (2009).

S3. M. E. Newman, *Proc. Natl. Acad. Sci. U. S. A* **103**, 8577 (2006).

S4. J. Reichardt, S. Bornholdt, *Phys. Rev. E. Stat. Nonlin. Soft. Matter Phys.* **74**, 016110 (2006).

S5. A. E. Teschendorff, M. Journee, P. A. Absil, R. Sepulchre, C. Caldas, *PLoS. Comput. Biol.* **3**, e161 (2007).
