# Supplementary material for: Role of DNA Methylation and Epigenetic Silencing of HAND2 in Endometrial Cancer Development
Source: PLoS Med. 2013 Nov 12;10(11):e1001551. doi: 10.1371/journal.pmed.1001551 (PMC3825654; doi:10.1371/journal.pmed.1001551)
Supplement: Text S2 — ARRIVE document. (DOC) [file pmed.1001551.s026.doc]

**Item 1**

**Title:** Conditional knockout of *Hand2* in uterine tissue leads to complex atypical hyperplasia as a function of age

**Item 2**

**Abstract:** Endometrial cancer is the other most common gynecologic disease worldwide. The molecular pathogenesis of this cancer remains unclear. Using an epigenome-wide methylation analysis, our recent studies identified HAND2 as one of the most common hypermethylated and silenced genes in endometrial cancer. To study the role of Hand2 in the development of endometrial cancers, we generated a mouse model in which Hand2 expression is conditionally ablated in uterine tissues and studied changes in endometrial histology as a function of age. Our results showed that the endometrium with loss of Hand2 expression developed precancerous lesions. The lesions appeared with increasing age and showed enhanced FGFR signaling and absent PTEN expression. Collectively, our studies revealed that HAND2 silencing is the most common molecular alteration in endometrial cancer and a crucial step in early carcinogenesis.

**Item 3**

**Introduction**

Background**:** Endometrial cancer is ten times more common in North America and Europe compared to less developed countries. This common gynecological disease is largely determined by age, obesity, reproductive and environmental factors. While etiology and pathogenesis of this cancer remains uncertain, it is generally believed that prolonged exposure of endogenous estrogen, exogenous xenoestrogen, or unopposed estrogen receptor (ER) signaling arisen from the defects in progesterone receptor (PR)-mediated action in the endometrium are among the major causal factors. Very little is known about early molecular changes, which contribute to the development of this disease. Our recent studies showed that Hand2 is a key mediator of PR signaling in mouse uterine stromal cells and play an essential role in inhibition of estrogen-stimulated uterine epithelial proliferation. Loss of Hand2 expression in uterine tissues leads to persistent epithelial proliferation. HAND2 is also expressed in the human endometrium at the mid-secretory phase of the menstrual cycle. Using an epigenome-wide DNA methylation approach, we identified HAND2 as one the most hyper-methylated and silenced gene in a large set of clinical uterine carcinoma samples. Since spatio-temporal expression and signaling of Hand2 is conserved in the mouse and the human endometrium, we reasoned that Hand2 conditional knockout mouse model will develop endometrial hyperplasia and provide an ideal system to study early events of the endometrial tumorigenesis.

**Item 4**

**Objective:** To study the role Hand2 in endometrial tumorigenesis using Hand2 conditional knockout mouse model.

**Item 5**

**Methods**

**Ethical statement:** All animal care, euthanasia, and tissue collection were strictly adhered to the National Institutes of Health and Institutional Guidelines for the use of laboratory animals. All animal protocols were reviewed and approved by the Institutional Animal Use and Care Committee (IAUCC) of the University of Illinois.

**Item 6**

**Study design:** To study the functional role of Hand2 in endometrial tumorigenesis, we generated a Hand2 conditional knockout mouse model by crossing Hand2 floxed mice (Hand2^f/f^) and PR-cre knock-in mice. Hand2 conditional knockout females (Hand2^d/d^) and the littermate controls (Hand2^f/f^) (N=24) were randomly divided into three groups and housed at 2-4 mice per cage. Uterine histology was assessed by H&E staining or immunohistochemistry at 8-10 (N=5 for each genotype), 24-32 (N=3 for each genotype) and 40-48 weeks of age (N=4 for each genotype), respectively.

**Item 7**

**Experimental procedures:** Hand2 conditional knockout mice (Hand2^d/d^) were generated by crossing Hand2^f/f^ mice with PR-cre knock-in mice (PR^cre/+^). Genotyping was performed based on standard protocol using purified mouse tail DNA. The Hand2^f/f^ PR^cre/+^ males and Hand2^f/f^ females were used as breeding pairs to generate Hand2 conditional knockout females (Hand2^d/d^) and the corresponding littermate controls (Hand2^f/f^). The number of animals per genotype per experiment was estimated according to the error variance. The mice were separated and randomly divided into three groups and housed at 2-4 mice per cage.

Female mice between 8-10, 24-32 and 40-48 weeks of age were euthanized by carbon dioxide inhalation at the designated space in the laboratory. Uterine tissues were excised, trimmed and collected. Uterine segments were fixed in 10% formalin fixative overnight, then in 75% ethanol for paraffin embedding. Paraffin-embedded endometrial tissues obtained from Hand2^d/d^ and Hand2^f/f^ were sectioned at 4 µm, mounted on slides and subjected to H&E staining or immunohistochemistry.

**Item 8**

**Experimental animals:** Hand2 floxed mice (Hand2^f/f^) are in C57BL/6 background and were generated in Dr. Deepak Srivastava’s laboratory at the University of California, San Francisco, California. PR-Cre knock-in mice are in 129Sv/C57BL/6 mixed background and were generated by Drs. John Lydon and Franco Demayo at the Baylor College of Medicine, Houston, Texas. All mice are tested for pathogen-free before housing.

**Item 9**

**Housing and husbandry:** Mice were maintained in an accredited animal care facility at the University Of Illinois, College Of Veterinary Medicine, according to the institutional guidelines for the care and use of laboratory animals. The animals were housed in an Allentown 140 cage IVC system set at 60 air changes per hour at the cage level. The mice were kept on Harlan 1/8 corncob and provided with Harlan 8604 rodent diet. Harlan Iso-blox was provided for environmental enrichment. Rooms were kept on a 12-12 light/dark cycle and the temperature was set at 72 degrees F with 35 ± 4% relative humidity. The water provided by the city was filtered and chlorinated. Mice were housed at 2-5 mice per cage and were provided with food and water. Pups were weaned by animal care staff or researchers at 21-days after delivery. Females and males were housed separately. Mice were monitored daily by trained animal care staff. Health status of the animals was assessed prior to, during, and after the experiments.

**Item 10**

**Sample size:** Uterine sections from Hand2^f/f^ and Hand2^d/d^ females at 8-10 (N=5 for each genotype), 24-32 (N=3 for each genotype) and 40-48 weeks of age (N=4 for each genotype) were subjected to H & E staining or immunohistochemical analysis.

**Item 11**

**Allocating animals to experimental groups:** Female mice of each genotype from different breeding pairs were randomly divided into three groups and uterine tissues were collected at designated ages.

**Item 12**

**Experimental outcomes:** Our studies revealed a marked increase in the gland-stroma ratio and an irregularity in the gland shape and size in uteri from Hand2^d/d^ mice compared to Hand2^f/f^ controls with increasing age. The endometrium of Hand2^d/d^ mice exhibited clear histological features of complex atypical hyperplasia including increased mitotic activity. We also observed that ablation of Hand2 in uterine tissue leads to molecular changes commonly observed in human endometrial cancer such as increased FGFR signaling and loss of Pten expression.

**Item 13**

**Statistical analysis:** The numerical values prepared from at least three independent samples were analyzed by t-test when comparisons were made between control and experimental groups (GraphPad Prism 4.0, GraphPad Software, Inc., San Diego, CA). Data are expressed as mean ± SEM. Significance is defined as P < 0.05.

**Results**

**Item 14**

**Baseline data:** Both Hand2^d/d^ and Hand2^f/f^ mice were healthy before tissue collection. No difference in appearances or body weights were observed between these two groups.

**Item 15**

**Numbers analyzed:** Uterine tissues from 24 females were analyzed.

**Item 16**

**Outcomes and estimation:** Uterine sections from both Hand2^d/d^ and Hand2^f/f^ females were subjected to H & E staining or immunohistochemical analysis using antibodies against cytokeratin 8, Ki67, p-FRS2, p53 or PTEN, respectively. Representative images from each group are shown. Staining with cytokeratin 8, a biomarker of epithelial cells, revealed closely packed irregular-shaped glands with multiple layers in uteri lacking Hand2. There is also an increase in the gland-stroma ratio in Hand2^d/d^ uteri compared to Hand2^f/f^ controls. The numbers of glands in more than three areas (4X microscope objective) in each of the uterine sections, in each group, were counted and expressed as mean +/- standard error. Our studies also revealed that the Hand2 null uteri exhibit a marked increase in phospho-FRS2 staining in the epithelium compared to floxed controls. While no alteration in p53 expression was observed between the two genotypes, we observed a lack of Pten expression in Hand2-null uteri.

**Item 17**

**Adverse events:** None observed.

**Discussion**

**Item 18**

**Interpretation/Scientific implication:** Our studies showed that loss of Hand2 in the uterus leads to morphological and distinct molecular changes, which precede invasive endometrial cancer. These results are consistent with our human data, which indicate that Hand2 is epigenetically silenced in the endometrium of patients with endometrial cancers. Collectively, these data support our hypothesis that Hand2 is a critical regulator of endometrial tumorigenesis.

**Item 19**

**Generalisability/translation:** Our studies using mice lacking uterine Hand2 clearly indicated that Hand2 plays a critical role in endometrial hyperplasia. Consistent with this observation, we found that Hand2 levels are reduced in human endometrial cancers. The Hand2 conditional knockout animal model, therefore, could serve as a critical resource to identify signaling pathways that are functioning downstream of Hand2 to regulate endometrial tumorigenesis.

**Item 20**

**Funding:** NIH grant U54 HD055787 as part of the *Eunice Kennedy Shriver* National Institute of Child Health and Human Development/NIH Centers Program in Reproduction and Infertility Research.
